# Copper depletion modulates mitochondrial oxidative phosphorylation to impair triple negative breast cancer metastasis

**DOI:** 10.1038/s41467-021-27559-z

**Published:** 2021-12-15

**Authors:** Divya Ramchandani, Mirela Berisa, Diamile A. Tavarez, Zhuoning Li, Matthew Miele, Yang Bai, Sharrell B. Lee, Yi Ban, Noah Dephoure, Ronald C. Hendrickson, Suzanne M. Cloonan, Dingcheng Gao, Justin R. Cross, Linda T. Vahdat, Vivek Mittal

**Affiliations:** 1 Department of Cardiothoracic Surgery, Weill Cornell Medicine, New York, NY 10065 USA; 2 Donald B. and Catherine C. Marron Cancer Metabolism Center, Memorial Sloan Kettering Cancer Center, New York, NY 10065 USA; 3 Department of Microchemistry and Proteomics, Memorial Sloan Kettering Cancer Center, New York, NY 10065 USA; 4 Department of Pharmacology, Weill Cornell Medicine, New York, NY 10065 USA; 5 Department of Biochemistry, Weill Cornell Medicine, New York, NY 10065 USA; 6 Department of Pulmonary and Critical Care Medicine, Weill Cornell Medicine, New York, NY 10065 USA; 7 The School of Medicine and Tallaght University Hospital, Trinity Biomedical Sciences Institute, Trinity College Dublin, Dublin, Ireland; 8 Department of Cell and Developmental biology, Weill Cornell Medicine, New York, NY 10065 USA; 9 Sandra and Edward Meyer Cancer Center, Weill Cornell Medicine, New York, NY 10065 USA; 10 Department of Medicine, Breast Medicine Service, Memorial Sloan Kettering Cancer Center, New York, NY 10065 USA

**Keywords:** Cancer metabolism, Breast cancer

## Abstract

Copper serves as a co-factor for a host of metalloenzymes that contribute to malignant progression. The orally bioavailable copper chelating agent tetrathiomolybdate (TM) has been associated with a significant survival benefit in high-risk triple negative breast cancer (TNBC) patients. Despite these promising data, the mechanisms by which copper depletion impacts metastasis are poorly understood and this remains a major barrier to advancing TM to a randomized phase II trial. Here, using two independent TNBC models, we report a discrete subpopulation of highly metastatic SOX2/OCT4+ cells within primary tumors that exhibit elevated intracellular copper levels and a marked sensitivity to TM. Global proteomic and metabolomic profiling identifies TM-mediated inactivation of Complex IV as the primary metabolic defect in the SOX2/OCT4+ cell population. We also identify AMPK/mTORC1 energy sensor as an important downstream pathway and show that AMPK inhibition rescues TM-mediated loss of invasion. Furthermore, loss of the mitochondria-specific copper chaperone, COX17, restricts copper deficiency to mitochondria and phenocopies TM-mediated alterations. These findings identify a copper-metabolism-metastasis axis with potential to enrich patient populations in next-generation therapeutic trials.

## Abstract (web summary)

Copper depletion has been reported to improve survival in patients with triple negative breast cancer (TNBC) but the underlying mechanisms are not completely understood. Here, the authors show that copper chelation reduces mitochondrial oxidative phosphorylation leading to decreased TNBC metastasis.

## Introduction

Triple-negative breast cancer (TNBC) is characterized by an absence of estrogen receptor (ER−), and progesterone receptor (PR−) expression, and also lacking overexpression of the HER2 epidermal growth factor receptor (HER2−). TNBC accounts for 10–15% of all breast cancers and is associated with poor outcomes due to a lack of response to current targeted therapies and higher rates of metastasis^1^. Given the limited availability of effective FDA-approved molecularly targeted anti-metastatic therapies, there is an unmet medical need for developing novel treatments for TNBC patients^2^. Metals function as co-factors for a host of metalloenzymes/proteins that contribute to tumor growth and metastasis and targeting metals to disable these pathways has emerged as a potential anti-cancer therapeutic strategy^3,4^. For example, Wilson’s disease, characterized by a mutation in the *ATP7B* gene, a copper-transporting P-type ATPase contributes to copper homeostasis by excreting excess intracellular copper into the bile and plasma. Mutations in *ATP7B* result in excess copper accumulation, which led to the development of tetrathiomolybdate (TM), a first-in-class oral copper chelating drug. TM was granted fast track designation by the FDA in 2017^5^ and is currently in phase III clinical trials for Wilson’s disease. Given the increased copper uptake by malignant cells^6^, cellular copper has emerged as a potential anti-cancer therapeutic target^7,8,9^.

Copper is an essential cofactor for a host of metalloenzymes/proteins including superoxide dismutase-1 (SOD1), vascular adhesion protein-1, MMP-9, lysyl oxidase, VEGF, and angiogenin^10–12^ that contribute to carcinogenesis^11,13–15^. Copper was shown to be a necessary binding partner for MEK1/2-mediated BRAF signaling in melanoma progression^16^, copper depletion was able to overcome resistance to BRAF and MEK1/2 inhibitors^17^, and regulate autophagy in lung adenocarcinoma^18^. In a model of colon cancer, inflammatory cytokines, such as IL-17 was shown to induce increased intracellular uptake of copper via increased expression of metalloreductase STEAP4, to promote tumorigenesis^19^. A recent study also demonstrated that copper chelators mediate ubiquitin-dependent PD-L1 degradation via inhibition of STAT3 and EGFR phosphorylation^20^. Copper chaperone, ATOX1, has been demonstrated as an important mediator of breast cancer metastasis, and therefore a potential biomarker for the effectiveness of copper depletion therapy^21^. In the Her2/neu breast cancer transgenic mice, TM treatment was associated with improved disease-free survival^22^. Our phase II clinical trial of TM (NCT00195091)^23^, with 75 breast cancer patients at high risk for relapse showed that TM was safe and well-tolerated with <3% reversible grade 3 or 4 adverse events. Importantly, this trial reported an event-free survival (EFS) of 72% and overall survival (OS) of 84% at a median follow-up of 6.3 years^23^. In this trial, the TNBC cohort with stage 4 disease showed EFS of 69% after 2 years; a striking finding as median EFS and OS of stage 4 TNBC patients is less than 8 months and 12 months, respectively, from multiple trials^24,25^. Advancing TM into larger randomized phase II trials will be facilitated by a better understanding of the mechanisms by which copper depletion impacts metastasis and may also inform patient selection for future trials.

Metabolic pathways that support cancer cell proliferation and tumor growth are under active investigation, however, less is known about their role in metastasis, in part because small numbers of disseminating tumor cells have been hard to investigate^26–28^. Cancer cells are highly glycolytic, even in the presence of oxygen, a phenomenon referred to as the “Warburg effect”^29^. Subsequently, high rates of both glycolysis and oxidative phosphorylation (OXPHOS) have been shown to be important for the production of biosynthetic intermediates necessary to support the rapid proliferation of both normal and cancer cells^30^. Indeed, recent studies have shown that certain tumors can also upregulate OXPHOS^31,32^, advocating the potential utility of OXPHOS inhibitors, such as metformin, as a potential therapeutic approach^33^.

In this work, we use in vivo limetastatic co/metastatic compartment in primary breast tumors, together with proteomic and metabolomic profiling to provide a direct link between copper-mediated metabolic reprograming and metastasis in TNBC.

## Results

### TM-mediated copper depletion reduces lung metastasis

**Fig. 1:**
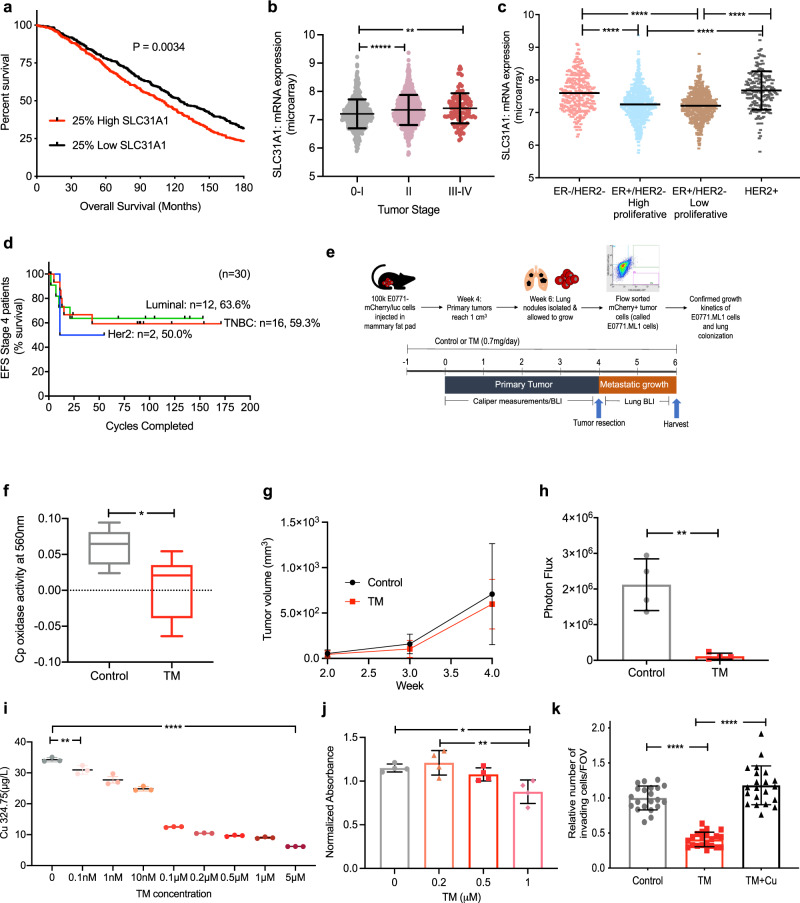
Copper depletion impacts invasion and metastasis. **a** Patient survival with CTR1 or SLC31A1 expression using METABRIC dataset. The graph depicts survival for patients with low expression (first quartile) and high expression (fourth quartile) of the *SLC31A1* gene (*n* = 425 patients/group). Significance was calculated using Log-rank (Mantel–Cox) test. **b** Distribution of *SLC31A1* expression with tumor stage. Significance was calculated using one-way ANOVA with Tukey post-test for comparing multiple groups. *N* = 433 for Stage 0-I patients, *n* = 702 for Stage II patients, *n* = 111 for Stage III–IV patients. *p*-value (0-I vs. II) < 0.0001, *p*-value (0-I vs. III–IV) = 0.0011. **c** Distribution of *SLC31A1* mRNA expression in breast cancer subtypes. Significance was calculated using one-way ANOVA with Tukey post-test for comparing multiple groups. *n* = 290 for ER−/PR− patients, *n* = 603 for ER+/HER2− High proliferative patients, *n* = 619 for ER+/HER2− Low proliferative patients, *n* = 188 for HER2+ patients. *p*-value < 0.0001. **d** Event-free survival (EFS) for Stage 4 NED breast cancer patients from TM clinical (Chan et al. 2017). 1 cycle = 4 weeks. **e** (Top panel) Development of EO771.ML1 model. (Lower panel) Timeline for metastasis assay with or without TM using EO771.ML1 model. **f** Ceruloplasmin levels in control vs. TM-treated mice in ML1 model, 2 weeks after TM administration (*n* = 6/group). Analysis was performed by unpaired two-sided *t*-test. Center lines of box plots denote median values, top whiskers denote maxima and bottom whiskers minima. *p*-value = 0.0224. **g** Primary tumor growth in control and TM cohorts in ML1 model, measured with calipers (*n* = 10 in the control group, *n* = 9 in TM group). Analysis was performed by unpaired two-sided *t*-test. *p*-value = 0.8365. **h** BLI measurements of lungs in ML1 model showing metastatic burden (*n* = 4/group). Analysis was performed by unpaired two-sided *t*-test. *p*-value = 0.0015. **i** Measurement of intracellular copper (Cu) in LM2 cells using graphite furnace atomic absorption spectrometry (*y*-axis refers to Cu content measured at 324.75 nm wavelength). Significance was calculated using one-way ANOVA with Tukey post-test for comparing multiple groups. p-value (0 vs. 0.1 nM) = 0.0003, *p*-value (rest) < 0.0001. **j** MTT assay for viability with increasing TM concentrations, 72 h after treatment of LM2 cells (*n* = 4/group). Significance was calculated using One-way ANOVA with Tukey post-test for comparing multiple groups. *p*-value (0 vs. 1 nM) = 0.0162, *p*-value (0.2 vs. 1 nM) = 0.0041. **k** Matrigel coated transwell assay showing invasion of LM2 cells treated with TM (0.5 µM) for a total of 72 h (48-h pretreatment, followed by 24 h treatment on Matrigel-coated transwells) and rescue with addition of copper (0.5 µM CuCl_2_) for the last 24 h in the transwell (along with TM for that cohort). *n* = 3 replicates/group (7 fields of view/sample). Significance was calculated using one-way ANOVA with Tukey post-test for comparing multiple groups. p-value (Control vs. TM + Cu) = 0.0115. Representative data of at least two independent experiments are depicted. Results are expressed as mean ± SD. *p < 0.05, **p < 0.01, ****p < 0.0001.

To determine the significance of copper in breast cancer, we examined the Molecular Taxonomy of Breast Cancer International Consortium (METABRIC) dataset of 2000 clinically annotated breast cancer patients^34^ for expression of the high affinity copper uptake protein 1 (CTR1), encoded by the *SLC31A1* gene. Patients with higher *SLC31A1* expression showed significantly poor overall survival (OS) compared to patients with low *SLC31A1* expression (Fig. 1a). Further analysis showed increased *SLC31A1* expression in patients with advanced/metastatic tumors (stage III–IV) (Fig. 1b). Additionally, patients with aggressive breast cancer subtypes: TNBC and HER2+ had higher *SLC31A1* expression than luminal type A or B subtypes (Fig. 1c). Therefore, these findings suggest that blocking copper uptake may be a therapeutic strategy in metastatic disease.

Our phase II clinical trial of TM (NCT00195091)^23^, showed no difference in outcomes among the different molecular subtypes in patients who were stage 4 NED (no evidence of disease). Indeed, at a median follow-up of 9.4 years, EFS for patients with the poorest prognosis was 63.6% for luminal breast cancers, 50% for Her2+ and 59.3% for TNBC (Fig. 1d). EFS of 59.3% was particularly striking in patients who had TNBC and were stage 4 without evidence of disease, as a median EFS of less than 8 months has been observed in previous trials^24,25^. These observations provide a strong rationale for the development of copper chelation as a therapeutic strategy in breast cancer patients with advanced disease. However, advancing TM into larger randomized phase II trials would be facilitated by a clearer understanding of the mechanistic basis by which copper depletion impairs metastatic progression, and this would also subsequently facilitate patient selection for TM treatment.

We previously showed that in an orthotopic model of TNBC (MDA-MB-231-LM2 or LM2), copper depletion did not impact primary tumor growth of cells, but significantly reduce lung metastases^23^. LM2 cells are a lung-tropic variant of the human MDA-MB-231 cell line which were selected for an enhanced ability to metastasize to lung tissue through two rounds of in vivo selection^35^. To extend these observations in a physiologically relevant, immunocompetent setting, we leveraged the syngeneic EO771 mouse model of TNBC containing homozygous mutations in Trp53 and KRAS^36^. This model is weakly metastatic to the lungs, but, following a similar procedure of in vivo selection, we generated a lung tropic variant cell line EO771.ML1 (ML1), after sorting mCherry+ cells from lung nodules (Fig. 1e, top panel). We then confirmed ML1 cells labeled with mCherry and firefly luciferase aggressively metastasized to lung tissue following orthotopic injection into the mammary fat pad.

Cohorts of tumor-bearing mice were treated with oral TM administered through drinking water (0.7 mg/day) (Fig. 1e, bottom panel). Copper levels were efficiently reduced in vivo, as determined by measurement of copper-carrying protein ceruloplasmin (Cp) in serum as described before^23^ (Fig. 1f). Primary breast tumors were surgically resected at 1 cm^3^, and after 2 weeks of primary tumor resection, lungs were analyzed for metastatic burden (Fig. 1e, bottom panel). TM treatment did not impair primary tumor growth (Fig. 1g); however, TM decreased lung metastatic burden, analyzed by whole-body BLI, 2 weeks after primary tumor resection (Fig. 1h). This demonstrates that copper depletion impairs metastatic disease progression even in the presence of an intact immune response.

To characterize the in vivo phenotypes, we treated LM2 cells in vitro with various concentrations of TM to identify the lowest TM dose that effectively depletes intracellular copper. Atomic absorption spectrometry showed a dose-dependent reduction in copper and TM at 10 nM and above efficiently depleted intracellular copper within 24 h of treatment initiation (Fig. 1i, Supplementary Fig. 1a) (30% of baseline levels consistent with patients in the TM trial). TM at concentrations of up to 0.5 µM did not impact cell viability (Fig. 1j) or proliferation (Supplementary Fig. 1b, c); however, the invasion was significantly reduced through a matrix-coated transwell (Supplementary Fig. 1d, e), a phenotype rescued by copper add-back (Fig. 1k). Together, these results suggest that TM-mediated loss of invasion may be the cause of reduced metastasis in vivo.

### Copper depletion selectively targets a unique population of highly metastatic SOX2/OCT4+ cells

**Fig. 2:**
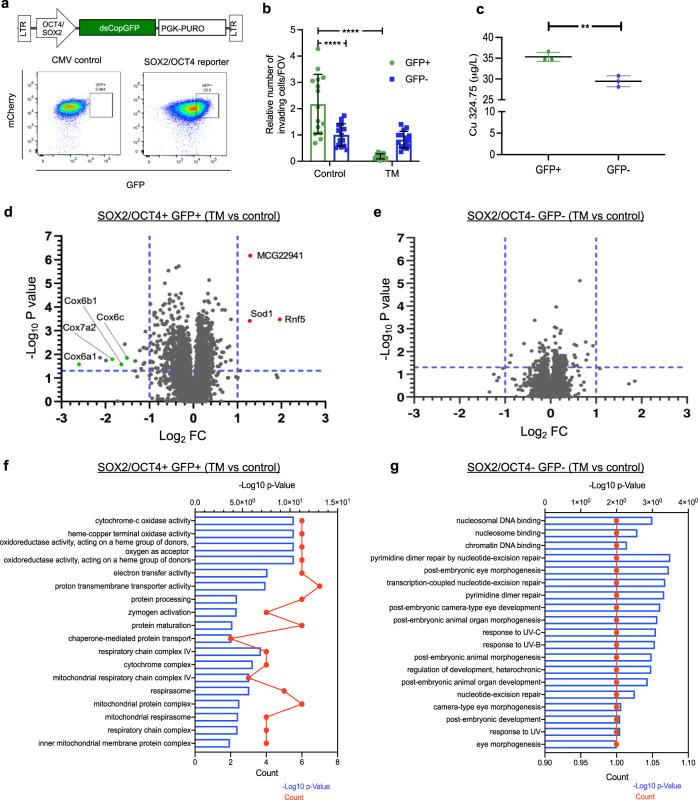
Copper depletion targets SOX2/OCT4+ metastatic cells. **a** (Top panel) schematic of lentivirus-based SOX2/OCT4-GFP promoter-reporter system. (Bottom panel) Flow-sorted GFP+ and GFP− cells from bulk ML1 cells with CMV control or SOX2/OCT4 reporter system. **b** Impact of TM (0.5 µM) on the invasion of SOX2/OCT4+ GFP+ and SOX2/OCT4− GFP− cells across a matrix coated transwell in LM2 cells. Significance was calculated using Two-way ANOVA (*n* = 3 replicates/group, 5 fields of view/sample). **c** Intracellular copper (Cu) levels in SOX2/OCT4+ GFP+ vs. SOX2/OCT4− GFP− cells measured by atomic absorption spectrometry (*y*-axis refers to Cu content measured at 324.75 nm wavelength) in ML1 cells. Analysis was performed by unpaired two-sided *t*-test. *p*-value = 0.0040. **d** Tandem mass tags (TMT) isobaric label multiplexed quantitative proteomic analysis of vehicle and TM (0.5 µM) treated ML1 SOX2/OCT4+ GFP+ reporter cells. Proteins with absolute log2 Fold Change (FC) > 1, *p* < 0.05 and FDR < 0.05 are shown in green and labeled, *n* = 5/group. Significance was calculated using multiple *t*-tests, *p* values were adjusted for multiple comparisons. **e** Tandem mass tags (TMT) isobaric label multiplexed quantitative proteomic analysis of vehicle and TM (0.5 µM) treated ML1 SOX2/OCT4− GFP− cells. Proteins with log2 > 1 or < −1 and *p* < 0.05 are shown, *n* = 5/group. Significance was calculated using multiple *t*-tests, *p* values were adjusted for multiple comparisons. **f** and **g** Gene ontology (GO) analysis of downregulated proteins (*p* < 0.05, average log2 < −0.6) after 48 h TM treatment in ML1 SOX2/OCT4+ GFP+ (**f**) and ML1 SOX2/OCT4− GFP− **g** cells (log2 fold change from −2.6 to −0.7). log2 fold change is log2 FC of TM 48 h/Control. Count refers to how many genes/proteins showed up in that GO term. GO analysis was performed using the clusterProfiler R package, *p* values were adjusted using Benjamin–Hochberg method. dsCop destabilized copepod. Representative data of two independent experiments are depicted. Results are expressed as mean ± SD. **p < 0.01 ****p < 0.0001.

Since TM impacted metastasis and not primary tumor growth, using the lentiviral SOX2/OCT4-GFP promoter reporter system, we lineage traced a subpopulation of SOX2+ OCT4+ cancer cells within the primary tumor, that exhibit stem cell phenotypes, and enhanced potential for metastasis in TNBC models^37,38^. We introduced the SOX2/OCT4-GFP reporter (Fig. 2a, top panel) into LM2 and ML1 cells and identified the GFP+ population (Fig. 2a, bottom right panel). As expected, lentivirus lacking the OCT4 and SOX2 response element (mini-CMVp-GFP) did not report these cells (Fig. 2a, bottom left panel). The sorted reporter positive (GFP+) cells were confirmed for expression of core stem cell transcription factors: SOX2 and OCT4, and their downstream target NANOG by qPCR (Supplementary Fig. 2a). As expected, GFP+ LM2 cells showed increased invasion in a transwell assay compared to GFP− cells, and TM treatment resulted in a dramatic reduction in the invasive potential of GFP+ cells, whereas the GFP− cells remained unaffected (Fig. 2b). To determine the source of enhanced sensitivity of the GFP+ cells to TM-mediated copper depletion, we first measured intracellular copper levels using atomic absorption spectrometry and found that GFP+ cells had higher basal intracellular copper levels, compared to GFP− cells (Fig. 2c).

Given that copper serves as an essential cofactor for a variety of proteins, we next performed a global analysis to determine the impact of TM-mediated copper depletion on this invasive/metastatic reporter-positive population. ML1 GFP+ and GFP− populations were sorted, treated with TM or vehicle for 48 hours, and quantitative protein abundance profiling was performed using liquid chromatography–mass spectrometry (LC–MS). This analysis identified differentially expressed proteins. Multiple subunits of mitochondrial Complex IV were specifically downregulated (log2 fold change < −1, *p* < 0.05, FDR < 0.05) after TM treatment in GFP+ cells compared to GFP− population (Fig. 2d, e). However, the proteomic analysis did not show differential expression of SOX2 and OCT4, possibly due to the low abundance of these transcription factors.

To perform gene ontology (GO) analysis of downregulated proteins, we analyzed the proteomics data with relaxed stringency (log2 fold change >0.6 or <−0.6 and *p* < 0.05). Analysis showed enrichment of pathways involved in the mitochondrial electron transport chain (ETC), specifically in ML1 GFP+ cells following TM treatment (Fig. 2f, g). Similarly, LM2 GFP+ and GFP− cells sorted from human LM2 cell line were also analyzed for quantitative protein abundance profiling performed using LC–MS following TM treatment for 48 h, and then GO analysis on downregulated proteins (log2 fold change < −0.6 and *p* < 0.05), revealed similar results for preferential enrichment of pathways associated with mitochondrial ETC in GFP+ cells (Supplementary Fig. 2b, c). Thus, TM treatment specifically impacted mitochondrial ETC pathways in SOX2/OCT4+ cells with higher intracellular copper levels.

### Copper depletion disrupts mitochondrial respiration in TNBC cells

Consistently, proteomic analysis of both human TNBC LM2 and murine ML1 models of TNBC identified pathways enriched in mitochondrial function (Supplementary Tables 1 and 2). Cytochrome *c* oxidase (Complex IV), a mitochondrial metalloenzyme, is the terminal enzyme of mitochondrial ETC. The catalytic core of cytochrome c oxidase is formed by three mitochondria-encoded subunits and contains three copper atoms^39^. Two copper atoms bound to subunit 2 constitute the Cu_A_ site, the primary acceptor of electrons from ferrocytochrome *c*. The third copper subunit, Cu_B_, is associated with the heme *a*_3_ group of subunit 1. In the absence of these copper centers, both subunits are rapidly degraded and the cytochrome c oxidase holoenzyme fails to assemble and function^39^. We, therefore, hypothesized that TM-mediated copper depletion led to the degradation of Complex IV subunits, detected in the proteomic analysis.

**Fig. 3:**
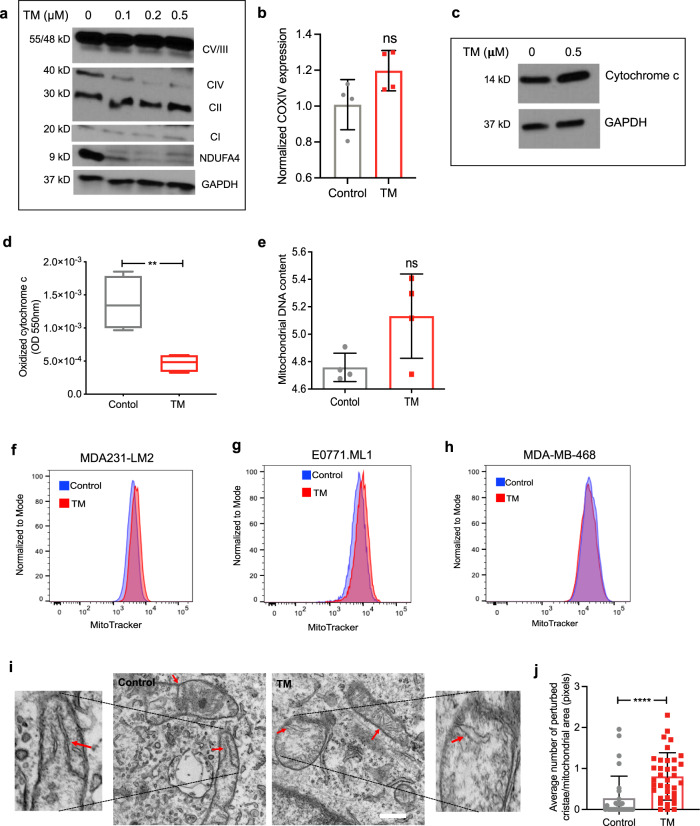
TM treatment impacts mitochondrial Complex IV. **a** Western blot showing respiratory complexes in LM2 cells following 72-h TM treatment (0, 0.1, 0.2, 0.5 µM). Complexes I–V are referred to as CI (mol. wt. ~19 kDa), CII (mol. wt. ~30 kDa), CIII (mol. wt. ~48 kDa), CIV (mol. wt. ~40 kDa), and CV (mol.wt. ~55 kDa), respectively. NDUFA4 is a subunit of Complex IV. **b** qPCR analysis showing transcript levels of human Complex IV subunit 4I1 (COX4I1) in control and TM (0.5 µM) treated LM2 cells, treated with TM for 72 h. Analysis was performed by unpaired two-sided *t*-test. *p*-value = 0.079. **c** Protein expression of cytochrome c with and without TM treatment (0.5 µM) (*n* = 4/group). **d** Human cytochrome c oxidase activity (Abcam) kinetically measured over 2 h after 72 h of treatment with TM (0.5 µM) in LM2 cells. The graph represents the difference in Complex IV activity at 110th minute during measurement. Analysis was performed by unpaired two-sided *t*-test (*n* = 4/group). *p*-value = 0.0063. Center lines of box plots denote median values, top whiskers denote maxima and bottom whiskers minima. **e** Mitochondrial DNA content in LM2 cells as determined by qPCR (*p* = 0.060). Analysis was performed by unpaired two-sided *t*-test (*n* = 4/group). **f–h** MitoTracker deep red analysis in control and TM (0.5 µM) treated LM2 (**f**), ML1 (**g**) and MDA-MB-468 (**h**) cells, treated for 72 h (*n* = 3/group). **i** Structure of cristae in the inner mitochondrial membrane as determined by transmission electron microscopy (×50,000), red arrows indicate normal/folded and perturbed cristae morphology in LM2 cells. Scale bar, 500 nm. **j** Quantification of perturbed cristae per 100 units of mitochondrial area in pixels (*n* = 30 mitochondria in controls, *n* = 36 mitochondria for TM group). Analysis was performed using Mann–Whitney two-sided test. Representative data of two independent experiments are depicted. Results are expressed as mean ± SD. **p < 0.01 ****p < 0.0001.

Western blot analysis using antibodies against ETC complexes confirmed a reduction in Complex IV subunits (Fig. 3a). As expected, copper depletion impacted protein levels of Complex IV without impacting its mRNA levels (Fig. 3b). TM-mediated loss of Complex IV subunits did not impact the expression of cytochrome c (Fig. 3c) but resulted in a significant reduction (>60%) in the oxidation of cytochrome c (Fig. 3d). We did not observe TM-mediated changes in mtDNA content (Fig. 3e) or in MitoTracker (deep red) fluorescence intensity as measured by flow cytometry (Fig. 3f–h). Further characterization of the mitochondrial phenotype using transmission electron microscopy showed that TM treatment perturbed the structure of mitochondrial cristae, which require intact ETC supercomplexes to maintain their shape^40^. We observed a significant increase in the number of perturbed cristae morphology in TM-treated cells (Fig. 3i, j). Together, these results suggest that TM-mediated alterations in mitochondrial structure and function may reprogram cellular bioenergetics.

### Copper depletion disrupts respiration in SOX2/OCT4-GFP reporter positive cells

**Fig. 4:**
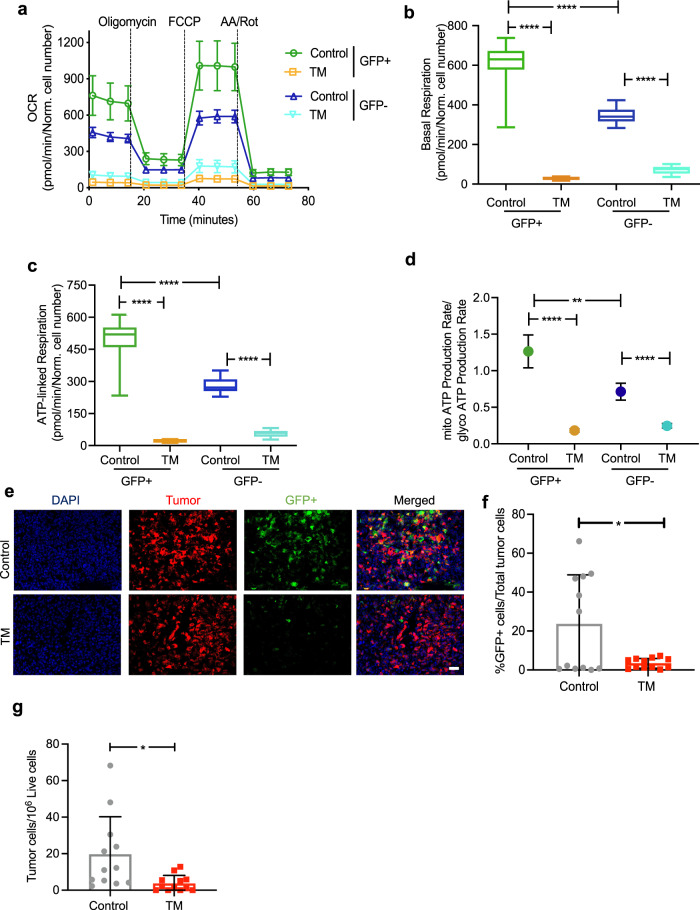
Copper depletion disrupts mitochondrial respiration in SOX2/OCT4+ metastatic cells. **a** Impact of TM (0.5 µM) on oxygen consumption rate of sorted GFP+ and GFP− ML1 cells as measured by Seahorse assay (*n* = 8/group). Quantification of basal respiration (**b**) and ATP-linked respiration (**c**) in GFP+ vs. GFP- ML1 cells with and without TM (0.5 µM). Significance was calculated using one-way ANOVA with Tukey post-test for comparing multiple groups. Center lines of box plots denote median values, top whiskers denote maxima and bottom whiskers minima. **d** Ratio of mitochondrial to glycolytic ATP production rate in GFP+ vs. GFP− ML1 cells, measured by Seahorse ATP production rate assay (*n* = 8/group). Significance was calculated using one-way ANOVA with Tukey post-test for comparing multiple groups. Representative data of two independent experiments are depicted. **e** Representative IF microscopy images from control and TM-treated (0.7 mg/day) primary LM2 tumors in the mammary gland (*n* = 5/group). Scale bar, 20 µM. **f** Flow cytometry analysis showing the percentage of GFP+ cells in primary tumors in the LM2 model (*n* = 12/group) of total mCherry+ tumor cells. Analysis was performed by unpaired two-sided *t*-test. *p*-value = 0.0111. **g** Disseminated tumor cells in early lungs of control and TM (0.7 mg/day) treated LM2 cohorts. Analysis was performed by unpaired two-sided *t*-test. *p*-value = 0.0150. Representative data of two independent experiments are depicted. Results are expressed as mean ± SD. Results are expressed as mean ± SD. **p < 0.01, ****p < 0.0001.

Extracellular flux analysis using Seahorse XF bioanalyzer showed that GFP+ cells exhibited a higher rate of oxygen consumption, as determined by Mitostress assay, compared to GFP- cells in both the ML1 (Fig. 4a) and LM2 model (Supplementary Fig. 3a). In the absence of TM treatment, GFP+ cells showed a 1.7-fold higher basal and ATP-linked respiration compared to GFP− cells (Fig. 4b, c). Notably, TM treatment resulted in a dramatic reduction in basal respiration in GFP+ ML1 (21-fold) cells compared to GFP− ML1 (5-fold) cells (Fig. 4b). Similarly, ATP-linked respiration was reduced in GFP+ ML1 cells (22-fold) compared to GFP− ML1 cells (5-fold) with TM treatment (Fig. 4c). After TM treatment, GFP+ cells had a 2.5-fold lower basal and ATP-linked respiration than TM-treated GFP− cells. We saw a similar pattern in the LM2 model (Supplementary Fig. 3b, c). Next, in the absence of TM treatment, we observed that GFP+ cells showed a 1.8-fold higher mitochondrial-to-glycolytic ATP production rate ratio compared to untreated GFP− cells (Fig. 4d). Additionally, the ratio of mitochondrial-to-glycolytic ATP production was markedly reduced in GFP+ cells by 6.6-fold after TM treatment, while in GFP− cells the reduction in mitochondrial-to-glycolytic ATP production rate ratio was only reduced by 1.6-fold following TM treatment (Fig. 4d). TM-treated GFP+ cells had a 1.3-fold lower mitochondrial-to-glycolytic ATP production rate ratio than TM-treated GFP− cells (Fig. 4d).

We have reported that the GFP+ cells exhibit increased dissemination from primary tumor, and when injected via tail vein, generate larger metastasis compared to GFP− cells^37^. Therefore, to determine if TM selectively targets GFP+ cells in vivo, we generated cohorts of mice with orthotopic immunocompetent ML1 and immunodeficient LM2 cells stably expressing the SOX2/OCT4-GFP promoter reporter system. Oral TM treatment reduced the percentage of GFP+ cells in the primary tumor, compared to control untreated mice as determined by microscopy imaging of tumor cells (mCherry+) and reporter positive GFP cells (Fig. 4e, Supplementary Fig. 3d). Consistent with microscopy analysis, flow cytometric analysis of primary tumors showed a marked reduction of GFP+ cells (Fig. 4f, Supplementary Fig. 3e). Gating strategy for in vivo LM2 and ML1 models is provided in Supplementary Fig. 4. TM treatment effectively reduced disseminated tumor cells (mCherry+) in early lungs (lungs harvested during primary tumor resection, without overt metastatic outgrowth) as determined by flow cytometry analysis (Fig. 4g). Taken together, these results suggest that copper depletion-mediated reduction in OXPHOS impairs invasion in vitro and targets a specific population of metastatic tumor cells in vivo and that TM targets a subpopulation of metastatic tumor cells that rely on OXPHOS.

Although TM-mediated copper depletion was achieved by 24 h (Supplementary Fig. 1a), we observed that the consequences of copper depletion on oxygen consumption rates were underdeveloped at 24-h and below (Supplementary Fig. 3f), and evident by 48 h and above (Supplementary Fig. 3g). This finding is consistent with the observation that copper depletion destabilizes Complex IV > 48 h after treatment (Supplementary Fig. 3h). Next, to determine if treatment with TM and copper depletion had induced a permanent metabolic alteration in the LM2 tumor cells, we removed TM after 48 h of treatment and observed that TM-mediated reduction in mitochondrial respiration had reverted to baseline levels, within 24 h, suggesting that the metabolic phenotype was reversible (Supplementary Fig. 3i, j). Additionally, oxygen consumption was also rescued by copper add-back to TM treated cells (Supplementary Fig. 3k). To validate TM-mediated metabolic phenotypes, we also used an independent copper chelator- trientine, which was able to efficiently reduce oxygen consumption (Supplementary Fig. 3l, m).

To confirm the pharmacological findings, we used a genetic model of global copper depletion. Using CRISPR/Cas9, we knocked-out the major copper transporter receptor 1 (CTR1)^41^ in LM2 cells (Supplementary Fig. 5a, b). Compared to control cells (SCR), CTR1 knockout (CTR1KO) cells were depleted of intracellular copper (Supplementary Fig. 5c), and consistent with TM phenotypes, showed a selective reduction in Complex IV (Supplementary Fig. 5d) and decreased mitochondrial respiration (Supplementary Fig. 5e), suggesting that genetic loss of CTR1 recapitulates TM phenotypes.

### Restricting copper deficiency to the mitochondrion phenocopies TM

Following copper uptake into the cells, copper-specific chaperones deliver copper to defined cellular compartments/proteins (e.g. ATOX1 transports copper to ATP7A and ATP7B in trans Golgi networks, CCS delivers to SOD1 in mitochondria, and COX17 delivers to Complex IV in mitochondria)^42^. Therefore, where the global copper depletion approach (TM and CTR1KO) may confound the direct role of copper-mediated mitochondrial bioenergetics on the loss of invasion and metastatic phenotypes, specifically restricting copper availability to mitochondria, will confirm copper depletion-mediated metabolic defects with metastasis.

**Fig. 5:**
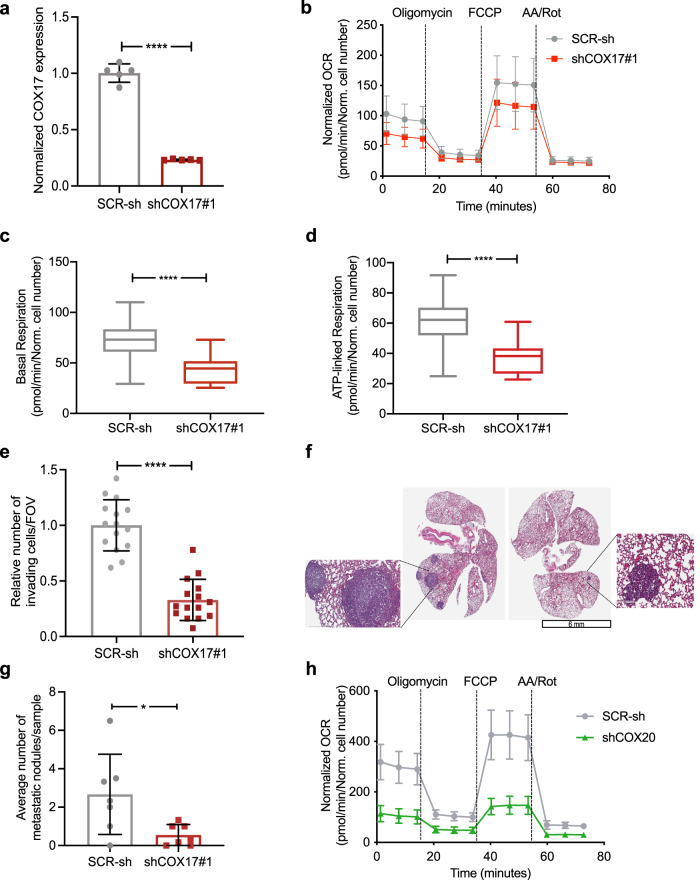
COX17 knockdown yields TM phenotypes. **a** qPCR showing shRNA-mediated COX17 knockdown in LM2 cells. Analysis was performed by unpaired *t*-test (*n* = 5/group). **b** Oxygen consumption in shCOX17#1 vs. SCR-sh (*n* = 8 for SCR-sh, *n* = 6 for shCOX17#1). **c**, **d** Basal and ATP-linked respiration in shCOX17#1 vs. SCR-sh cells using mitostress assay. Analysis was performed by unpaired two-sided *t*-test. Center lines of box plots denote median values, top whiskers denote maxima and bottom whiskers minima. **e** Invasion assay through matrix-coated transwell. Analysis was performed by unpaired two-sided *t*-test (*n* = 3/group, 5 fields of view/sample). **f** Representative images of metastatic nodules in lungs using shCOX17#1 and SCR-sh LM2 in vivo model (*n* = 7/group). **g** Quantification of lung metastases from panel **f**. Analysis was performed by unpaired two-sided *t*-test. *p*-value = 0.0236. **h** Oxygen consumption rate in ML1 SCR-sh vs. shCOX20 cells (*n* = 8/group). Representative data of two independent experiments are depicted. Results are expressed as mean ± SD. *p < 0.05, ****p < 0.0001.

To limit copper delivery to the mitochondria, we targeted cytochrome c oxidase 17 (COX17) that specifically transports copper to Complex IV^43^. COX17 knockdown with two independent shRNAs (shCOX17#1, shCOX17#2) efficiently reduced COX17 expression (Fig. 5a, Supplementary Fig. 6a). Knockdown of COX17 (shCOX17) reduced oxygen consumption and ATP-linked respiration compared to scrambled controls (SCR-sh) (Fig. 5b–d, Supplementary Fig. 6b). Transwell invasion assay showed decreased invasion in shCOX17 cells compared to SCR-sh (Fig. 5e, Supplementary Fig. 6c). To confirm the specificity of COX17 knockdown, we generated silent mutations in the shCOX17 binding site (Supplementary Fig. 6d), confirmed its refractoriness to knockdown by shCOX17 (Supplementary Fig. 6e). Expression of mutant COX17 rescued invasion defects mediated by shCOX17 (Supplementary Fig. 6f). As expected from the invasion phenotypes, COX17 knockdown reduced lung metastases in vivo (Fig. 5f, g). Taken together, these results suggest that restricting copper availability specifically to the mitochondria elicits a significant metabolic, invasion, and anti-metastatic phenotype, consistent with global copper chelation. To directly demonstrate that Complex IV dysfunction is the main cause for the observed mitochondrial phenotypes, we also perturbed COX20 in ML1 cells, an assembly factor for Complex IV^44^. Indeed, shRNA-mediated COX20 knockdown (Supplementary Fig. 6g) resulted in reduced oxygen consumption (Fig. 5h, Supplementary 6h) and impaired invasion through a matrigel coated-transwell (Supplementary Fig. 6i). Together, these results suggest that Complex IV dysfunction resulting from either mitochondrion-specific copper depletion or direct targeting of COX20 phenocopies TM-induced metabolic defects.

### Copper depletion activates AMPK to inhibit mTORC1 signaling

**Fig. 6:**
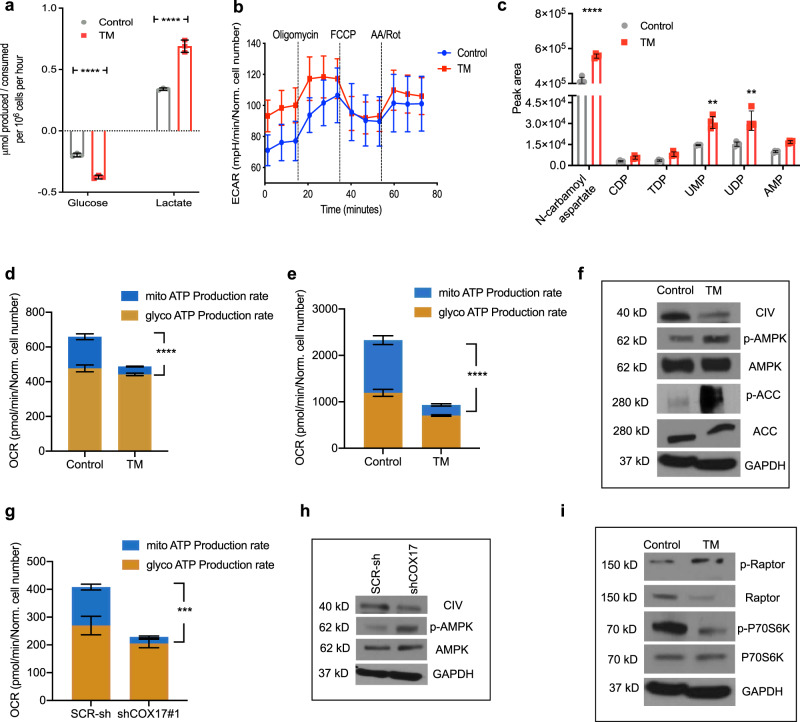
Copper depletion activates AMPK. **a** Glucose consumption and lactate production measured by nutrient analysis after 72 h of TM (0.5 µM) treatment in LM2 cells. Supernatant was replaced 24-h before collection in both groups. Significance was calculated using Two-way ANOVA (*n* = 3/group). **b** ECAR for LM2 cells after 72 h of TM (0.5 µM) treatment (*n* = 8/group). **c** Levels of nucleotide precursors measured by LC–MS after 72 h of TM (0.5 µM) treatment in LM2 cells. Significance was calculated using Two-way ANOVA, followed by Bonferroni’s multiple comparisons test (*n* = 5/group). **d** and **e** Mitochondrial ATP production rate with TM (0.5 µM) treatment in LM2 (**d**) and ML1 (**e**) cells. Significance was calculated using Two-way ANOVA, followed by Tukey’s multiple comparisons test (*n* = 8/group). **f** Western blot of p-AMPK (Thr172), p-ACC (Ser79), and Complex IV (CIV) in control and TM (0.5 µM) treated LM2 cells. Complex IV is referred to as CIV. **g** Mitochondrial ATP production rate in LM2 SCR-sh and shCOX17#1, using Seahorse ATP production rate assay. Significance was calculated using Two-way ANOVA, followed by Tukey’s multiple comparisons test (*n* = 3 for SCR-sh, *n* = 8 for shCOX17#1). **h** Western blot of p-AMPK in shCOX17 and SCR-sh LM2 cells. Complex IV is referred to as CIV. **i** mTOR pathways (p-Raptor Ser792, p-p70S6K Thr389) downstream of phospho-AMPK in LM2 cells. Representative data of two independent experiments are depicted. Results are expressed as mean ± SD. **p < 0.01, ***p < 0.001, ****p < 0.0001.

We posited that TM-mediated reduction in OXPHOS may reprogram cells to upregulate glycolysis. Nutrient analysis in LM2 cell supernatants demonstrated that treatment with TM increased glucose consumption and lactic acid production (Fig. 6a), a phenotype supported by the higher basal extracellular acidification rate (Fig. 6b) observed in LM2 cells.

Coincidentally, TM increased levels of nucleotide precursors (Fig. 6c), including AMP, compared to untreated controls, as observed by metabolite profiling. Copper-dependent Complex IV participates in the formation of the mitochondrial transmembrane potential which is used by the mitochondrial F_1_F_o_-ATPase to drive ATP synthesis^45^. Therefore, we assessed the impact of TM on ATP production. As expected, TM significantly reduced mitochondrial (oxidative) ATP production, compared to glycolytic ATP production in both human and murine TNBC cells (Fig. 6d, e). Given that AMP is a major trigger for activation of the metabolic sensor AMP-activated protein kinase (AMPK)^46^, and could accumulate if not utilized for ATP production, we posited that copper depletion may have induced the activation of AMPK. We observed that TM-mediated copper depletion in LM2 cells increased AMPK phosphorylation at Thr172 (Fig. 6f), with a concomitant reduction in Complex IV (Fig. 6f), indicating activation of AMPK and phosphorylation of the downstream enzyme acetyl CoA carboxylase (ACC) at Ser79 was also increased (Fig. 6f). Consistent with TM, COX17 knockdown also showed decreased mitochondrial ATP production (Fig. 6g) and increased phospho-AMPK (Fig. 6h) levels. AMPK activation is known to inhibit the mammalian target of rapamycin complex 1 (mTORC1) pathway^47,48^. Consistent with this, TM increased phosphorylation of raptor (Ser792) (Fig. 6i), and reduced phosphorylation of mTORC1 downstream target P70S6K (Thr389) (Fig. 6i). TM increased the phosphorylation of 4E-BP1 (Supplementary Fig. 7)^49,50^. Collectively, these data suggest that TM-mediated disruption of the ETC creates bioenergetic stress which results in downstream activation of AMPK and reduced activity of mTORC1.

### AMPK activation reduces invasion

**Fig. 7:**
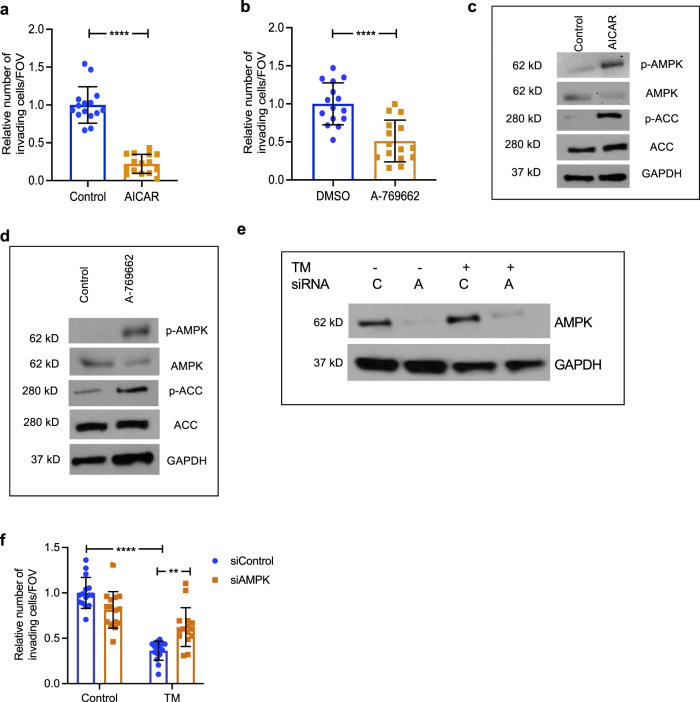
AMPK is a mediator of downstream TM phenotypes. **a**, **b** Impact of pharmacologic activation of AMPK with AICAR (1.25 mM) and A-769662 (12.5 µM) on the invasion of LM2 cells. Representative data from two independent experiments. Analysis was performed by unpaired two-sided *t*-test (*n* = 3/group, 5 fields of view/sample). **c** Western blot showing activation of AMPK by AICAR (1.25 mM) in LM2 cells. **d** Western blot showing activation of p-ACC by A-769662 (12.5 µM) in LM2 cells. **e** Control and 0.5 µM TM-treated cells (after 24 h) were transfected with siRNA against a control (C) sequence or AMPK (A). AMPK expression by western blot in LM2 cells. **f** Rescue of TM-mediated loss of invasion with siAMPK in LM2 cells. Significance was calculated using Two-way ANOVA, followed by Tukey’s multiple comparisons test (*n* = 3/group, 5 images/sample). Representative data of two independent experiments are depicted. Results are expressed as mean ± SD. **p < 0.01, ****p < 0.0001.

AMPK is a known regulator of energy homeostasis, cell growth, and autophagy; however, more recent studies have implicated AMPK signaling in cell migration and invasion^51,52^. To determine if AMPK activation contributes to decreased invasion, we used pharmacological AMPK activators: AICAR (adenosine analogue 5-aminoimidazole-4-carboxamide riboside) and A-769662^53^. Treatment of LM2 cells with either drug resulted in a significant reduction in invasion (Fig. 7a, b). As expected, there was increased phosphorylated AMPK and phosphorylated ACC after treatment with both drugs (Fig. 7c, d), thus confirming target engagement. To determine if TM-mediated defects in the invasion were mediated through AMPK activation, we used siRNA to inhibit the expression of AMPK (Fig. 7e) and observed that AMPK inhibition rescued TM-mediated loss of invasion phenotype (Fig. 7f). Together these results demonstrate that AMPK is a downstream mediator of TM-mediated invasion defects.

## Discussion

Copper depletion has emerged as a viable cancer therapeutic approach in breast cancer due to increased cellular uptake by malignant cancer cells and reliance on copper-dependent signaling pathways^11,54,55^. Indeed, our analysis of the METABRIC dataset^34^ showed breast cancer patients with high *SLC31A1* expression were associated with poor OS compared to patients with low *SLC31A1* expression. In line with these findings, recent analysis (median follow-up of 9.4 years) of data from our phase II clinical trial of TM^23^, showed a striking EFS of 59.3% in patients with stage 4 TNBC. To accelerate the design of larger randomized TM trials in TNBC, our goal was to identify mechanisms by which copper depletion impacts metastasis. By conducting TM dose response assays, we identified the lowest dose of TM that effectively depletes intracellular copper to approximately 30% baseline levels as achieved in the TM clinical trial^23^. Such an approach ensures that copper-dependent malignant cells are impacted, but normal copper-dependent physiological functions are maintained. Possibly, TM administered at higher concentrations by virtue of depleting copper to non-physiological levels has the potential to reduce cell viability, proliferation and increase apoptotic cell death^56,57^. We show that TM as low as 0.5 µM effectively depleted intracellular copper and did not impact cell proliferation or viability in vitro or primary tumor growth in vivo (at a dose of 0.7 mg/day). However, it significantly reduced cell invasion in vitro, and metastatic dissemination, and over all lung metastatic burden in vivo thus supporting the role of TM as a potent anti-metastatic agent^22^.

To directly establish a link between TM-mediated copper depletion and metastasis in TNBC, we deployed the SOX2/OCT4 promoter reporter lineage tracing system, which we recently reported marks a discrete subpopulation of cancer cells in the primary tumor with increased invasive and metastatic potential^37^. Notably, the SOX2/OCT4+ reporter positive metastatic cells exhibited elevated basal intracellular copper levels and exhibited marked sensitivity to TM compared to the reporter negative cells. Global proteomic and metabolomic profiling identified TM-mediated inactivation of copper-dependent mitochondrial Complex IV as the primary metabolic defect in the SOX2/OCT4+ population. TM-mediated destabilization of Complex IV was associated with a significant reduction in the oxidation of cytochrome c that plays a key role in mitochondrial bioenergetics. The mitochondria in TM treated cells showed perturbed (unfolded or improperly folded) cristae morphology, consistent with the observations that cristae shape affects the overall performance of electron transport chain, energy production and metabolic adaptations, making them “bioenergetic membrane of the mitochondrion”^40^.

While there has been significant progress in understanding the contribution of metabolic reprogramming to tumor growth, the role of metabolism in facilitating metastasis is poorly understood^26^. Indeed, the SOX2/OCT4+ cells with high intracellular copper exhibited elevated OXPHOS compared to SOX2/OCT4− cells, a finding consistent with the notion that tumors are metabolically heterogeneous, and that cancer stem cells with high metastatic and tumorigenic potential are more reliant upon OXPHOS than the bulk tumors^58,59^. Indeed, copper enriched SOX2/OCT4+ cells were markedly sensitive to copper depletion, as inactivation of Complex IV significantly reduced OXPHOS and increased glycolysis resulting in impaired invasion and metastatic phenotypes. Together, these findings provide the most direct evidence linking copper-induced metabolic reprograming and metastasis in TNBC. Copper is an essential cofactor for a host of metalloenzymes/proteins that contribute to carcinogenesis^10,11,60,61^, so it is conceivable that copper depletion is likely to elicit context-dependent effects based on tumor type and organ-specific microenvironments. Indeed, copper chelation in the RIP-Tag model of pancreatic cancer, expressing the SV40T oncogene under control of the rat insulin promoter, and in human endometrial cancer cells resulted in Complex IV inactivation and HIF-1α stabilization^56,62^ or inhibition of SOD1^63^. Similarly, copper depletion was able to overcome resistance to BRAF and MEK1/2 inhibitors^17^, and regulated autophagy in lung adenocarcinoma^18^.

While our study uncovers that copper depletion by altering metabolic reprogramming of select population of SOX2/OCT4+ metastatic cells that reside in the primary tumor, as a potential antimetastatic therapeutic strategy, it has been reported that in the context of metastasis, copper also serves as a critical co-factor for lysyl oxidase that has been implicated in the establishment of the premetastatic niche^61^. Hypoxic primary breast tumors secrete lysyl oxidase-like 2 (LOXL2), a copper-dependent amine oxidase^64^ that crosslinks collagen with elastin to generate a stiffened extracellular matrix (ECM), to generate “pre-metastatic niches” that support metastatic colonization and outgrowth^61,65,66^. Indeed, we previously reported that copper depletion impacts TNBC metastasis through inactivation of lysyl oxidase in preclinical models, and notably, in the phase 2 TM trial, serum LOXL2 was reduced over time in patients who were copper depleted^23,64^.

Given the various functions of intracellular copper, global copper depletion approach (TM and CTR1KO) has the potential to confound the direct role of copper-mediated mitochondrial bioenergetics on loss of invasion and metastatic phenotypes. To limit copper delivery to mitochondria, we targeted the mitochondrial copper chaperone, COX17, that transports copper to Complex IV^43^. Notably, mitochondria-specific copper restriction by COX17 knockdown phenocopied TM-mediated metabolic defects, and these results were further confirmed by direct targeting of COX20, a key assembly factor for Complex IV. TM-mediated reduction in OXPHOS decreased ATP peak area as determined by metabolite profiling. Whether this reduction is due to any changes in the rates of nucleotide synthesis with copper depletion would need to be confirmed with a tracing experiment, such as 13C or 2H serine for dTMP synthesis or 13C asparagine for aspartate. As a consequence of reduced ATP, TM treatment triggered the AMPK-mTORC1 pathway^46^. Given that in our study, copper depletion impacted invasion/metastasis phenotypes, we particularly focused on AMPK, which has been extensively studied in the context of energy homeostasis, cell growth and autophagy^48,51,67^. However, there are conflicting reports on the role of AMPK in cell migration, invasion and metastasis^68–70^. We showed that treatment with TM, reduced phosphorylation of mTORC1 downstream target P70S6K as a result of AMPK activation, which has also been implicated in regulation of actin cytoskeleton^67^, and was shown to be susceptible to inhibition by rapamycin^71^. By demonstrating that AMPK inhibition rescues TM-mediated loss of invasion phenotypes, our study provides the first evidence that links copper depletion-mediated AMPK activation to TNBC invasion and metastasis.

Tumor metabolism has emerged as an attractive target for therapy development, and several drugs including metformin (mitochondrial respiration), enasidenib (mutated IDH2), l-asparaginase, devimistat (PDH/αKGDH), CB-839 (glutaminase inhibitor) are being used in various cancer trials^72–75^. However, drugs targeting altered metabolism in metastasis have not been elucidated. Our findings identify a copper-metabolism–metastasis axis and highlight its potential to be developed as a next-generation therapeutic approach for high-risk TNBC patients.

## Methods

### Gene expression database and Phase II TM study

METABRIC dataset for breast cancer patients (EGAS000000000083) in cBioportal contains gene expressions for about 2000 patients performed on the Illumina HT-12 v3 platform^34^. Using this cohort, the expression of major copper transporter, CTR1, was analyzed for association with patient survival and tumor stage (0–I, II, III–IV). Also, tumor samples were assigned to intrinsic breast cancer subtypes: TNBC (ER−/HER2−), ER+/HER2− (high or low proliferative) and ER−/HER2+. Following this, the expression of CTR1 was analyzed within the breast cancer subtypes. Using data from our previously published phase II TM study (NCT00195091) in high-risk breast cancer patients^23^, we calculated EFS after 13 years in NED patients, continuing TM treatment. The study was conducted under Weill Cornell Medicine protocol #0309006307 and #0611008853, and under Memorial Sloan Kettering Cancer Center protocol #18-023, and appropriate informed consent was obtained from patients.

### Cell lines, cell culture, and DNA constructs

MDA-MB-231.LM2 (LM2) were obtained from Dr. Joan Massagué (MSKCC) and cultured as described^37,38^. EO771.ML1 (ML1) was generated from parental EO771 cells^36^. EO771 cells, expressing mCherry and firefly luciferase, were injected in the mammary gland, and matched metastatic lesions from lungs were harvested and sorted for mCherry+ cells to generate metastatic variant ML1, first derivative of lung metastatic cells.

Copper-depleting agents: TM and Trientine were purchased from Sigma Aldrich. TM was stored in dark, at −20 °C under argon to maintain stability. Cell viability was tested by MTT assay by plating cells in 5 replicates in 96-well plates and enumerating viability daily for 3 days. Cell cycle analysis was performed by propidium iodide staining (Abcam) according to the manufacturer’s protocol and manual cell counting was also assessed over a period of 72 h. Human COX17 (hairpin #1: TRCN0000046062, hairpin #2: TRCN0000046060) and mouse COX20 (TRCN0000339218) knockdown was achieved using lentiviral shRNA vectors from the TRC shRNA collection (Sigma-Aldrich, St. Louis, MO). AMPK activation was induced by 5-Aminoimidazole-4-carboxamide 1-β-d-ribofuranoside (AICAR, 1.25 mM; Sigma) and A-769662 (12.5 µM; ApeXbio). CRISPR/Cas9 knockout of CTR1 was performed using guide sequence (TTGGTGATCAATACAGCTGG, BROAD Institute GPP sgRNA designer) by electroporation of CrisprCas9 RNP, using the Neon Transfection System (Thermofisher Scientific). SOX2/OCT4-GFP lentiviral promoter-reporter was used to generate immunodeficient LM2 and immunocompetent ML1 stable cell lines using puromycin selection as described^37^. All lentivirus supernatants were generated in HEK-293T cells (ATCC).

### In vivo tumor growth and FACS analysis

All animal work was conducted in accordance with protocols and ethical guidelines approved by the Institutional Animal Care and Use Committee at Weill Cornell Medical College (WCMC). The mice were housed at 12 h/12 h dark/light cycle, with room temperature 65–75°F and 40–60% humidity. For the syngeneic ML1 model, one cohort of 8-week old female C57Bl/6j mice (Jackson Laboratory) was administered TM for 1 week before orthotopic tumor implantation. After 1 week, 1 × 10^5^ ML1 cells in 50 μL HBSS were injected into the fourth mammary fat pad of the control and TM-treated group. Primary tumors were allowed to grow for 4 weeks, or until 1 cm^3^, following which they were resected, and monitored for development of lung metastases weekly, for an additional two weeks after resection. Lung metastases were measured by bioluminescence imaging (BLI). For in vivo tumor growth using a human xenograft model, 8-week-old SCID mice (Charles River) were injected in the fourth mammary fat pad with 1 × 10^6^ LM2 cells in 50 μL PBS. Tumor growth was monitored weekly by BLI and caliper measurements. At 6 weeks of tumor growth, or until 1 cm^3^, mice were euthanized by IP injection of ketamine/xylazine, followed by cervical dislocation for harvesting primary tumors or lungs. Tissue for immunofluorescence analysis was washed, fixed, and embedded in OCT. For histological analysis, tissues were paraffin-embedded and used for H&E staining. For flow cytometry analysis, tissues (primary tumor or lungs) were diced, ground through a 140 μm mesh, filtered through a 70 μm filter, red blood cells were lysed, followed by live/dead staining using Zombie Aqua, and Fc blocked and surface stained with human CD44 (clone BJ18) and mouse CD45 (clone 30-F11) antibodies as described before^38^. Disseminated tumor cells in the lungs were identified as CD45− CD44+ mCherry+ per 10^6^ live cells. Human LM2 and mouse ML1 cells stably expressing the SOX2/OCT4−GFP promoter-reporter were identified by FACS as CD45− CD44+ mCherry+ GFP+ and CD45− mCherry+ GFP+, respectively. Analysis of flow cytometry data was performed using FlowJo v 10.6.2.

### Copper measurements with graphite furnace atomic absorption spectrometry (GFAAS)

LM2 and ML1 cells were treated with vehicle (water) or TM (0.5 µM). 2 × 10^6^ or 4 × 10^6^ cells were digested overnight with 50% HNO_3_ + 0.01% digitonin at 65 °C. Digested cells were used to measure copper content with Pinnacle 900z GFAAS machine (Perkin Elmer) against copper standards.

### Invasion Assays

For transwell assays, cells (25,000 cells for bulk LM2 or FACS sorted SOX2/OCT4− GFP− and SOX2/OCT4+GFP+; 40,000 for MDA-MB-468; 60,000 cells for ML1) were plated in triplicates onto the top chamber of 6.5 mm inserts, coated with Matrigel (Corning #356231), in 24-well plates with serum-free DMEM. The cells were treated with TM or vehicle for 48 h before plating the cells for invasion. The bottom chamber contained complete DMEM media with 10% FBS. The cells migrated for 24 h. After migration, the insert was washed twice with fresh PBS and fixed using Kwik-Diff™ Staining kit (ThermoFisher), according to the manufacturer’s protocol. After staining, images were obtained using a computerized Zeiss microscope (Axiovert 200 M) and analyzed using Axiovision 4.6 software (Carl Zeiss Inc.). For assays using AICAR (1.25 mM) and A-769662 (12.5 µM), cells were either pre-treated with a drug or control for 24 h before plating for invasion. For rescue experiments with copper, cells were treated with 0.5 µM CuCl_2_ for at least 2 h before plating the cells in the top chamber and continued with CuCl_2_ treatment for the remainder of the experiment. For rescue experiments with siAMPK (SCBT) or siControl (SCBT), cells were either treated with TM (0.5 µM) or not (controls) for 24 h prior to transfecting siAMPK (10 nM) or siControl (10 nM) using Lipofectamine3000. Cells (25,000) were plated on Matrigel-coated inserts for invasion assay, 48 h after transfection with siAMPK or siControl in the presence or absence of TM. Inserts were stained after 24 h of plating.

### LC–MS proteomic analysis

Cultured cells were treated with or without TM for 48 h. After treatment cells were trypsinized, washed with PBS twice and quantitative protein abundance profiling was performed using 16plex TMT chemical labeling and MS3, multi-notched LC–MS analysis on at least 3–5 replicates for each sample using Orbitrap Fusion^76^. Cell pellets were lysed in 8 M Urea, 200 mM EPPS (4-(2-Hydroxyethyl)-1-piperazinepropanesulfonic acid), pH 8.5 with protease (complete mini EDTA-free, Roche) and phosphatase inhibitors (cocktail 2 and 3, Sigma). Samples were then sonicated (Diagenode Bioruptor) for 3 cycles (1 min ON/1 min OFF). BCA assay was used to determine the protein concentrations. Aliquots of 100 µg were taken for each sample (based on BCA assay) and reduced with 5 mM TCEP (tris(2-carboxyethyl) phosphine hydrochloride), alkylated with 10 mM IAA (iodoacetamide), and quenched with 10 mM DTT (dithiothreitol). Samples were diluted to 100 µL with lysis buffer and precipitated by chloroform–methanol^77^. Pellets were resuspended in 50 µL 200 mM EPPS buffer, digested with Lys-C protease at a 1:50 protease-to-protein ratio for 5 h at 37 °C, then overnight with trypsin (1:50) at 37 °C.

Anhydrous acetonitrile was added at a final volume of 30%. TMTPro (16-plex) reagents were added to peptides at a 2.8:1 (TMT reagent-to-peptide ratio) and incubated for 1 h at room temperature. A label check was performed to determine mixing ratios, labelling efficiency, and number of missed cleavages by pooling 1 µg from each sample, desalting, then analyzing by mass spectrometry. Samples were mixed 1:1 across all channels, dried to remove acetonitrile, then desalted using C18 solid-phase extraction (SPE) Sep-Pak (Waters), and vacuum centrifuged to dryness. Dried samples were then immediately fractionated by high pH. Fractions (flow-through and washes) were vacuum centrifuged and reconstituted in 0.1% formic acid (FA) for LC–MS/MS.

Samples were reconstituted in 1 mL of 2% ACN/25 mM ABC. Peptides were fractionated into 48 fractions. An Ultimate 3000 HPLC (Dionex) coupled to an Ultimate 3000 Fraction Collector using a Waters XBridge BEH130 C18 column (3.5 um 4.6 × 250 mm) was operated at 1 mL/min. Buffer A consisted of 100% water, buffer B consisted of 100% acetonitrile, and buffer C consisted of 25 mM ABC. The fractionation gradient operated as follows: 1% B to 5% B in 1 min, 5% B to 35% B in 61 min, 35% B to 60% B in 5 min, 60% B to 70% B in 3 min, 70% B to 1% B in 10 min, with 10% C the entire gradient to maintain pH. The 48 fractions were then concatenated to 12 fractions (i.e., fractions 1, 13, 25, 37 were pooled, followed by fractions 2, 14, 26, 38, etc.) so that every 12th fraction was used to pool. Pooled fractions were vacuum-centrifuged then reconstituted in 1% ACN/0.1% FA for LC–MS/MS.

Fractions were analyzed by LC–MS/MS using a nanoAQUITY UPLC (Waters) with a 50 cm (inner diameter 75 µm) EASY-Spray Column (PepMap RSLC, C18, 2 µm, 100 Å) heated to 60 °C coupled to an Orbitrap Fusion Lumos Tribrid Mass Spectrometer (Thermo Fisher Scientific). Peptides were separated at a flow rate of 300 nL/min using a linear gradient of 1–35% acetonitrile (0.1% FA) in water (0.1% FA) over 4 h and analyzed by SPS-MS3. MS1 scans were acquired over a range of *m*/*z* 375–1500, 120K resolution, AGC target of 4 × 10^5^, and maximum IT of 50 ms. MS2 scans were acquired on MS1 scans of charge 2–7 using isolation of 0.7*m*/*z*, collision-induced dissociation with activation of 35%, turbo scan and max IT of 50 ms. MS3 scans were acquired using specific precursor selection (SPS) of 10 isolation notches, *m*/*z* range 100–1000, 50K resolution AGC target of 1e^5^. Differentially expressed proteins (log2 fold change>1 or <−1 and *p* < 0.05) were identified.

### TMT data analysis

Raw data files were processed using Proteome Discoverer (PD) version 2.4.1.15 (Thermo Scientific). For each of the TMT experiments, raw files from all fractions were merged and searched with the SEQUEST HT search engine with a Mus musculus/ Homo sapiens UniProt protein database downloaded on 2019/12/13 (92,249 entries). Methionine oxidation was set as variable modification, while cysteine carbamidomethylation, TMT16plex (K), and TMT16plex (N-term) were specified as fixed modifications. The precursor and fragment mass tolerances were 10 ppm and 0.6 Da, respectively. A maximum of two trypsin missed cleavages were permitted. Searches used a reversed sequence decoy strategy to control peptide false discovery rate (FDR) and 1% FDR was set as the threshold for identification. Gene ontology analysis was performed on significantly downregulated proteins using mouse genome for ML1 cells. The count refers to gene/protein count.

### Immunoblot and qPCR

Cell lysates were prepared using RIPA buffer (Millipore) with the addition of PMSF and protease inhibitor. The proteins were separated on a 4–15% SDS polyacrylamide gel electrophoresis, transferred on PVDF membranes and blocked with 5% non-fat milk. The membranes were probed with the required antibodies: OXPHOS antibody cocktail (Cat# ab110413, abcam), NDUFA4 (Cat# BS3883, Bioworld antibodies), AMPK (Cat# 5831S, CST), p-AMPK (Cat# 50081S, CST), ACC (Cat# 3662S, CST), p-ACC (Cat# 3661S, CST), Raptor (Cat# 2280S, CST), p-Raptor (Cat# 2083S, CST), P70S6K (Cat# 9202S, CST), p-P70S6K (Cat# 9204S, CST), p-4E-BP1 (Cat# 2855T, CST), 4E-BP1 (Cat# 9644T, CST), CTR1 (Cat# 13086S, CST), α-tubulin (Cat# 11224-1-AP, Proteintech) or GAPDH (Cat# ab9485, abcam).

For qPCR, total RNA was extracted using RNAeasy Plus (Qiagen) according to manufacturer’s protocol. qPCR primers for human Complex IV subunit 4I1 (Forward: CATGTGGCAGAAGCACTATGTGT; Reverse: GCCACCCACTCTTTGTCAAAG), human COX17 (Forward: CGATGCGTGTATCATCGAGAA; Reverse: TCATGCATTCCTTGTGGGC), mouse COX20 (Forward: CTTACAAGGTTCCACTGTAGGTAT; Reverse: TGAAGTTGCTTAGCTGCTGTTG), mouse SOX2 (Forward: AAGAAAGGAGAGAAGTTTGGAGCC; Reverse: GAGATCTGGCGGAGAATAGTTGG), mouse OCT4 (Forward: AGAACATGTGTAAGCTGCGG; Reverse: AGAACATGTGTAAGCTGCGG), mouse NANOG (Forward: AGGGTCTGCTACTGAGATGCTCTG; Reverse: CAACCACTGGTTTTTCTGCCACCG), human SOX2 (Forward: TAAATACCGGCCCCGGCGGA; Reverse: TGCCGTTGCTCCAGCCGTTC), human OCT4 (Forward: TGCCGTTGCTCCAGCCGTTC; Reverse: AAATAGAACCCCCAGGGTGAGC), human NANOG (Forward: ACATGCAACCTGAAGACGTGT; Reverse: CATGGAAACCAGAACACGTGG), human GAPDH (Forward: CTTCAACAGCGACACCCACTCCTC; Reverse: GTCCACCCTGTTGCTGTAG), mouse actin (Forward: TGAGCTGCGTTTTACACCCT; Reverse: TTTGGGGGATGTTTGCTCCA) and mouse GAPDH (Forward: TCACCACCATGGAGAAGGC; Reverse: GCTAAGCAGTTGGTGGTGCA) were obtained from IDT.

### Cytochrome c oxidase activity

For checking the activity of Complex IV of cytochrome c oxidase, we used a human Complex IV enzyme activity microplate assay kit (Abcam, ab109909). An equal number of LM2 cells (control vs. 0.5 µM TM-treated for 72 h) were lysed according to the manufacturer’s protocol and whole-cell lysates were used to determine Complex IV activity. Briefly, the enzyme was captured in the wells of the microplate and the activity was determined by oxidation of reduced cytochrome c (substrate) colorimetrically by the following absorbance at 550 nm, every 2 min over a period of 2 h using a plate reader. The difference in the activity between control and TM treatment was measured using the first and 110th-minute reading.

### Mitochondrial number

Mitochondrial numbers per cell were determined by both qPCR and MitoTracker deep red. For mtDNA content determination using qPCR, following primers for human cell line were used: mtDNA1 (16S rRNA Forward: GCCTTCCCCCGTAAATGATA; Reverse: TTATGCGATTACCGGGCTCT), nDNA β2-microglobulin, ß2M (Forward: TGCTGTCTCCATGTTTGATGTATCT; Reverse:

TCTCTGCTCCCCACCTCTAAGT). mtDNA content was calculated as described previously^78^, using the formula:

1 $$(\beta 2{{{{{\rm{M}}}}}}\,{{{{{\rm{average}}}}}}\,{{{{{{\rm{C}}}}}}}_{{{{{{\rm{T}}}}}}})-({{{{{{\rm{mtDNA}}}}}}}^{16{{{{{\rm{SrRNA}}}}}}}\,{{{{{\rm{average}}}}}}\,{{{{{{\rm{C}}}}}}}_{{{{{{\rm{T}}}}}}})=\varDelta {{{{{{\rm{C}}}}}}}_{{{{{{\rm{T}}}}}}}$$

2 $$2\times 2(\varDelta {{{{{{\rm{C}}}}}}}_{{{{{{\rm{T}}}}}}})={{{{{\rm{mtDNA}}}}}}\,{{{{{\rm{content}}}}}}$$

Mitochondrial content was also determined using MitoTraker deep red (used at 50 nM), according to manufacturer’s protocol, via flow cytometric analysis.

### Electron microscopy

Triplicates of control and TM-treated cells cultured in monolayers in a six-well plate, were fixed with 4% PFA, 2.5% glutaraldehyde, and 0.002% picric acid in 0.1 M sodium cacodylate buffer, pH 7.3 overnight at 4 °C. Samples were washed with buffer and post-fixed in aqueous 1% OsO_4_, 1.5% potassium ferricyanide for 1 h, washed with buffer. After a wash in deionized water, the samples were en bloc stained with 1.5% uranyl acetate(aqueous) for 1 h in the dark. Samples were dehydrated through a graded ethanol series then infiltrated with epoxy resin (LX112, Ladd Research Industries). Polymerized blocks were trimmed and sectioned at 65 nm. Sections were mounted on 200 mesh, thin-bar copper grids and post-stained with lead citrate. Samples were viewed on a JEOL JSM 1400 electron microscope (JEOL, US, Peabody, MA) and images were captured on a Veleta 2K × 2K CCD camera (EMSIS, GmbH, Muenster, Germany). Quantification of abnormal cristae/mitochondrial area was done using ImageJ software.

### Seahorse assays

Oxygen consumption rate (OCR) and extracellular acidification rate (ECAR) were measured using a Seahorse XFe96 Analyzer. Pre-treatments with TM were performed in 10 cm plates. For both MDA-MB-231-LM2 and EO771.ML1 20,000 cells were plated per well of the 96-well XFe plate 24 h prior to the assay. LM2 and ML1 cells were then analyzed using Seahorse XFe96 Analyzers according to the manufacturer’s protocol. Seahorse Wave Desktop Software (v2.6.1) was used for measurements. Oligomycin (oligo) and carbonyl cyanide 4-(trifluoromethoxy) phenylhydrazone (FCCP) concentrations were optimized and adjusted for each cell line (1 µM oligo and 1 µM FCCP was used for LM2, and 2 µM oligo and 0.5 µM FCCP was used for ML1 experiments). Basal respiration, ATP-linked respiration, proton leak, maximal respiration, spare capacity, and non-mitochondrial respiration were then calculated from the OCR Mitostress test profile. Differential glycolytic and mitochondrial ATP production rate was measured in a seahorse assay, using an ATP rate assay kit, according to the manufacturer’s protocol. At the end of each assay, total cellular DNA content was quantified, and acquired values were used for OCR and ECAR values normalization. Briefly, media was aspirated, and cells were stained overnight with 0.5% methylene blue. The next day, methylene blue was replaced with 4% acetic acid in 40% methanol, and after 10 min absorbance was measured at 668 nm.

### Measurement of glucose consumption and lactate secretion

Cells were plated in six‐well cell culture plates at a concentration aimed to reach 0.5–1 × 10^6^ cells at the time of harvest and treated with TM (0.5 µM) for 48 h. Media were exchanged for an assay period of 24 h (±TM) in the last 24 h of the treatment period, then collected, centrifuged, and analyzed using a 2950 Biochemistry Analyzer (YSI Life Sciences) to determine glucose, and lactate concentration. Absolute rates of consumption/secretion of these metabolites were calculated by subtracting the concentration in medium incubated for the same amount of time without cells, then normalizing to the cell number at the time of harvest, media volume, and hours of incubation.

### Metabolite profiling by LC–MS/MS

LM2 cells were plated in five replicates for each treatment, where at the end of the treatment period (72 h), there were approximately a million cells per well. At the end of treatment, cell number was determined from a single well for each condition, and four remaining replicates were used for metabolites extraction. Cells were washed with PBS (1×, w/o Ca^2+^/Mg^2+^), and metabolites were extracted with cold 80% methanol (1 mL/well). After overnight incubation at −80 °C, samples were vortexed and centrifuged at 20,000 × *g* for 20 min to remove protein. Supernatants were dried in a vacuum evaporator (Genevac EZ-2 Elite) for 3 h. For metabolomic profiling dried extracts were resuspended in 50 μL of 97:3 water:methanol containing 10 mM tributylamine and 15 mM acetic acid. Samples were vortexed, incubated on ice for 20 min, and clarified by centrifugation at 20,000 × *g* for 20 min at 4 °C. Ion pair LC–MS analysis was performed with LC separation on a Zorbax RRHD Extend-C18 column (150 mm × 2.1 mm, 1.8 μm particle size, Agilent Technologies), and using a gradient of solvent A (10 mM tributylamine and 15 mM acetic acid in 97:3 water:methanol) and solvent B (10 mM tributylamine and 15 mM acetic acid in methanol) according to the manufacturer’s instructions (MassHunter Metabolomics dMRM Database and Method, Agilent Technologies). The acquisition was performed using an Agilent 6470 LC/MS/MS triple quadrupole. MasHunter Workstation Software for Quantitative analysis B.09 (Agilent Technologies), RStudio v1.3. and GraphPadPrism v8 and v9 were used for data analysis.

### Immunostaining and microscopy

For immunofluorescence staining, 10 μM-thick tumor sections were air-dried in dark at room temperature, followed by blocking and staining with DAPI (1:1000). Fluorescent images for DAPI (nuclei), GFP+ (SOX2/OCT4 reporter), and mCherry (tumor cells) were obtained using a computerized Zeiss fluorescent microscope (Axiovert 200 M), fitted with an apotome and an HRM camera. Images were analyzed using Axiovision 4.6 software (Carl Zeiss Inc.).

### Histo-pathologic analysis for lung metastases

1 × 10^6^ LM2 cells with shCOX17#1 or SCR-sh were injected in the fourth mammary fat pad of 8-week-old female SCID mice. When primary tumors reached 1 cm^3^, they were surgically removed, and metastases were allowed to grow in the lungs of these mice for another 2 weeks. Two weeks after surgery, lungs from these mice were harvested, fixed in formaldehyde (4%), dehydrated with ethanol, embedded in paraffin, and then slides containing 5 μm sections were prepared. The slides were subsequently stained with hematoxylin and eosin Y solution (H&E) to assess histological alterations via light microscopy, scanned by Aperio, and analyzed for tumor nodules using ImageScope.

### Site-directed mutagenesis of shCOX17-binding sites in COX17

Site-directed mutagenesis was performed for shCOX17#2 hairpin using Q5^®^ Site-Directed Mutagenesis Kit (NEB) and the following primers: mutantF (5′-cccgaaagccaagaaaaaaagccgctgaagccc-3′) and mutantR (5′-cggggcagggtttgagtcaaccag-3′) for inducing silent mutations in wild-type COX17 (NM_005694) Human Tagged ORF Clone (Origene) to make it resistant to shCOX17#2. This shCOX17#2 mut (6.25 ng) was transfected into LM2 cells stably expressing shCOX17#2 using lipofectamine and used for invasion assays.

### Statistical analysis

Experiments contained positive and negative controls and were repeated for reproducibility and statistical rigor. Two independent TNBC models (human and murine) were used. Copper chelation was performed using two independent drugs TM (0.5 µM) and trientine (50 µM), and pharmacological findings were confirmed with precise genetic approaches. Statistical analyses were performed using an unpaired *t*-test with a normality test. Any outlier, if at all, was excluded after analyzing the datasets with the ROUT method. *T*-test was used to analyze significance between two groups. If the data did not pass the normality test, the Mann–Whitney test was used to calculate significance between two groups. One-way ANOVA with Tukey post-test was used for comparing three or more groups. For comparing metabolites, two-way ANOVA was used. *p* values < 0.05 were considered statistically significant. Results are expressed as mean ± SD unless otherwise mentioned.

### Reporting summary

Further information on experimental design is available in the Nature Research Reporting Summary linked to this paper.

## Supplementary information

Supplementary Information

Reporting summary

## Data Availability

The authors declare that the data supporting the findings of this study are available within the paper [and its Supplementary Information files]. Source data are provided with this paper. Raw proteomics data have been uploaded to ProteomeXchange.org (Project accession: PXD027089). Other datasets used in the study include: METABRIC (EGAS00001001753, https://www.cbioportal.org/study/summary?id=brca_metabric) and Phase II clinical trial of TM (NCT00195091) (Chan et al., 2017). Any additional detail can be requested from the corresponding authors. Source data are provided with this paper.

## Source data

Source Data
