# Supplementary material for: Copper depletion modulates mitochondrial oxidative phosphorylation to impair triple negative breast cancer metastasis
Source: Nat Commun. 2021 Dec 15;12:7311. doi: 10.1038/s41467-021-27559-z (PMC8674260; doi:10.1038/s41467-021-27559-z)
Supplement: Supplementary file 1 — Supplementary Information [file 41467_2021_27559_MOESM1_ESM.pdf]

SUPPLEMENTARY TABLE

Supplementary Table 1. Proteins differentially regulated in human LM2 TNBC cell line, with 48 hours of TM (0.5 µM) treatment. Quantitative protein abundance profiling was performed using 16-plex TMT chemical labeling and MS3, multi-notched LC-MS analysis on at least 5 replicates for each sample using Orbitrap Fusion. Significance was calculated using multiple t-tests, p values were adjusted for multiple comparisons. Highlighted proteins in green are subunits of mitochondrial Complex IV.

**Protein**	**Localization**	**Avg log2 LM2 +TM/-TM**	**Adj. P-Value**
ITFG2	cytosol, membrane	-2.89	2.59E-04
COX2	membrane, mitochondrion	-2.02	1.08E-02
NDUFA4	membrane, mitochondrion	-1.3	2.65E-08
APOC3	endosome, extracellular	-1.27	6.81E-05
TTN	chromosome, cytosol, extracellular	-1.24	8.39E-10
HBA2, HBA1	cytosol, extracellular, membrane	-1.12	9.31E-07
COX6A1	membrane, mitochondrion	-1.1	8.11E-04
APOB	cytosol, endoplasmic reticulum, endosome, extracellular, membrane	-1.02	1.07E-09
DHFR, DHFRP1	cytosol, mitochondrion	-1.01	1.00E-09
C12orf57	cytosol	-1	2.16E-04
SULT1A4	cytoplasm, cytosol	1	2.30E-06
VCAM1	endoplasmic reticulum, endosome, golgi, membrane	1.05	1.84E-09
PLAUR	extracellular, membrane	1.06	3.89E-07

HLA-DRB3	membrane	1.07	2.12E-06
CD99L2	membrane	1.08	2.48E-06
HLA-DRA	cell surface, membrane, vacuole	1.09	4.76E-06
SOD2	mitochondrion	1.1	4.41E-07
CPVL		1.1	2.65E-08
HLA-DQA1	membrane	1.14	6.78E-09
MFI2, MELTF	extracellular, membrane	1.15	1.13E-06
PHB	cytoplasm, endosome, membrane, mitochondrion, nucleus	1.17	3.66E-01
FBXO2	cytosol, endoplasmic reticulum, membrane	1.19	3.84E-05
ITGA1	membrane	1.25	3.75E-09
KYNU	cytosol, mitochondrion	1.27	8.39E-10
SOD1	cytosol, extracellular, membrane, mitochondrion, nucleus, vacuole	1.35	1.02E-04
HLA-DPA1	membrane	1.53	2.17E-06
ICAM1	membrane	1.78	1.66E-06
CA9	membrane, nucleus	1.87	8.39E-10
ICAM1	membrane	2.17	4.81E-07
CST1	extracellular	2.22	5.14E-06
IL8, CXCL8	extracellular	2.26	4.43E-08
IL1B	cytosol, extracellular, vacuole	2.32	1.21E-07
PTGS2	cytosol, endoplasmic reticulum, membrane, nucleus	3.31	9.82E-09

Supplementary Table 2. Proteins differentially regulated in mouse ML1 TNBC cell line, with 48 hours of TM (0.5 μM) treatment. Quantitative protein abundance profiling was performed

using 16-plex TMT chemical labeling and MS3, multi-notched LC-MS analysis on at least 5 replicates for each sample using Orbitrap Fusion. Significance was calculated using multiple t-tests, p values were adjusted for multiple comparisons. Highlighted proteins in green are subunits of mitochondrial Complex IV.

**Protein**	**Localization**	**Avg log2 ML1 +TM/-TM**	**Adj. P-Value**
A2MG	extracellular	-2.89	1.57E-01
FGG	extracellular	-2.55	1.00E+00
COX2	mitochondrion	-2.31	2.06E-05
COX6B1	mitochondrion	-1.63	2.06E-05
CD37	plasma membrane	-1.56	5.25E-02
FN1	extracellular	-1.34	1.00E+00
COX7A2	mitochondrion	-1.30	3.42E-03
COX6c	mitochondrion	-1.21	6.06E-07
FGB	cytosol, extracellular	-1.10	1.00E+00
NCOA4	lysosome, mitochondrion	-1.08	1.40E-01
ITIH2	extracellular	-1.06	2.50E-01
NDUFA4	mitochondrion	-1.00	3.28E-05
IRF7	cytosol, nucleus	1.05	4.81E-01
K1C13	cytoskeleton, nucleus	1.07	1.00E+00
UBP18	cytosol, nucleus	1.19	6.08E-01

SUPPLEMENTARY FIGURES

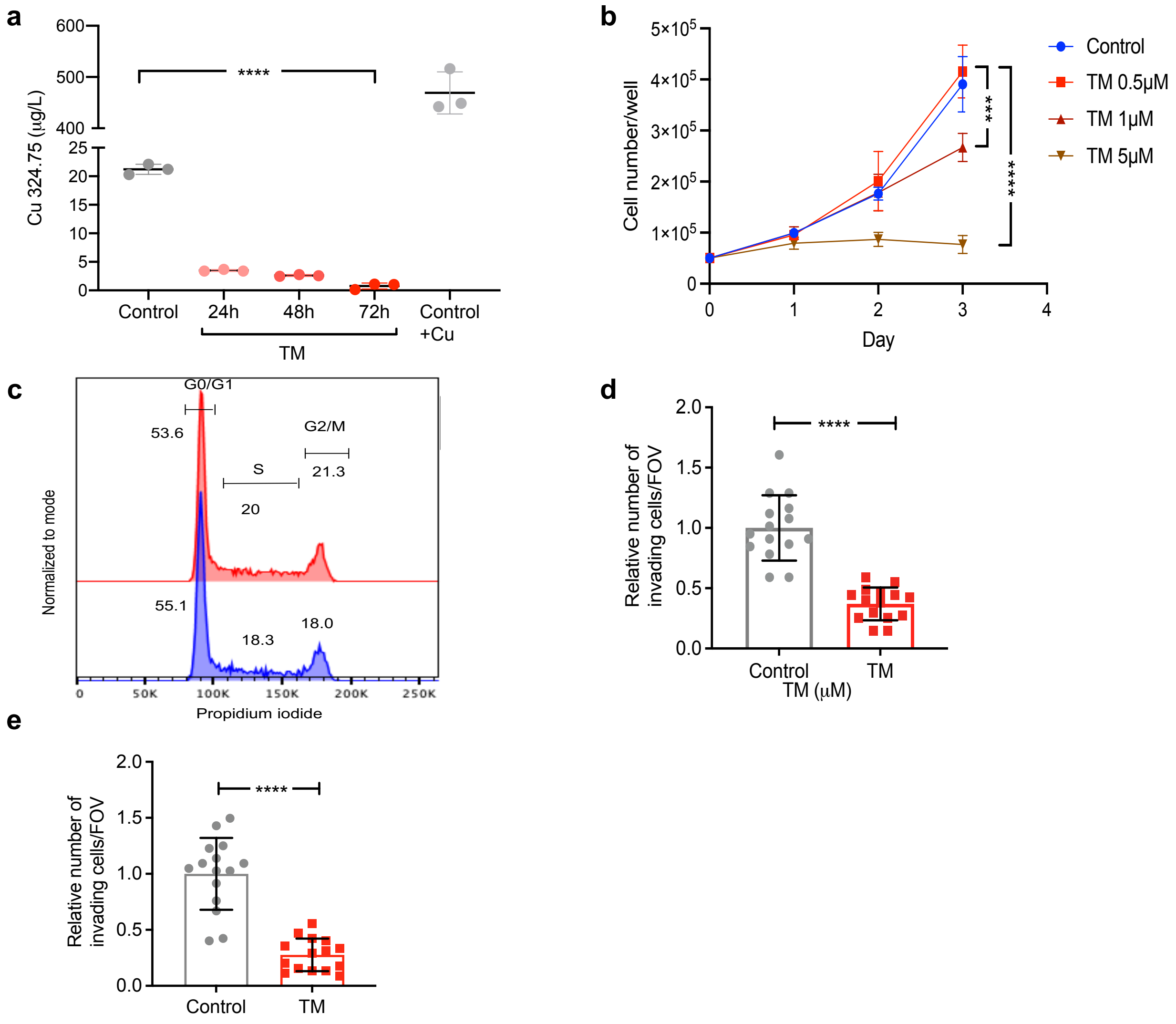

Supplementary Figure 1. (a) Time course analysis of intracellular copper content measured by atomic absorption spectrometry following treatment with TM (0.5 µM) for 72 hours (y-axis refers to Cu content measured at 324.75 nm wavelength) in LM2 cells. 3 repeated measures of the same sample. Significance was calculated using One-way ANOVA with Tukey post-test for comparing multiple groups. **(b)** Cell proliferation rate as measured by cell counting in LM2 cells. Significance was calculated using two-way analysis of variance (ANOVA) with Tukey's post hoc test. (n=3 replicates/sample). **(c)** Cell cycle distribution of LM2 cells with and without TM at 0.5 µM using propidium iodide staining, after 72 hours of treatment with TM (n=3 replicates/sample). Effect of

TM on invasion in MDA-MB-468 **(d)** and ML1 **(e)** cells (n=3 replicates/group, 5 fields of view/sample). Analysis was performed by unpaired two-sided t-test. Representative data of two independent experiments are depicted. Results are expressed as mean ± SD. ***p<0.001, ****p<0.0001

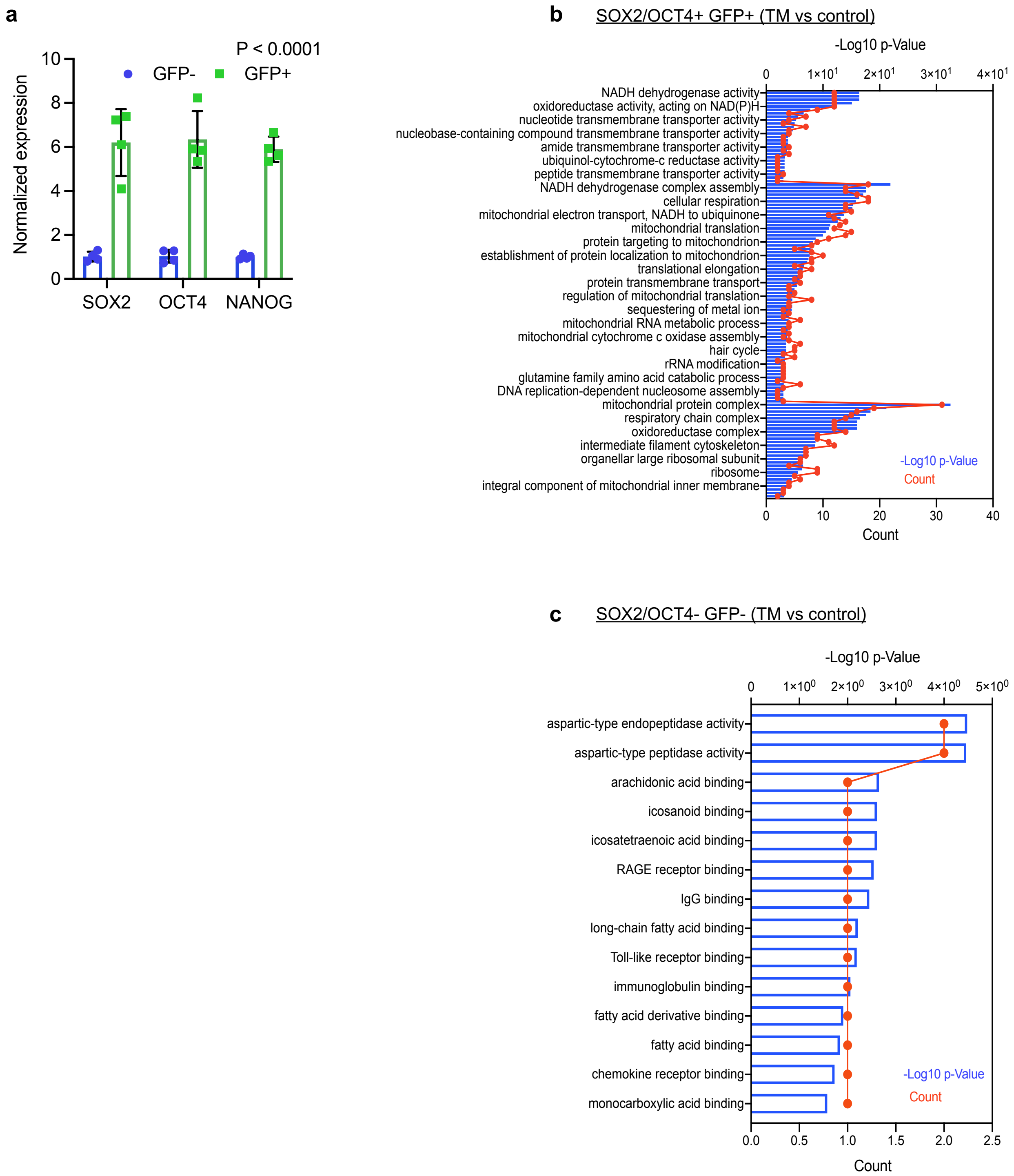

Supplementary Figure 2. (a) qPCR expression levels of SOX2, OCT4 and NANOG in sorted GFP- and GFP+ ML1 cells used in proteomic analysis. Data was analyzed using two-way ANOVA. Results are represented as mean ± SD (n=4/group). p-value<0.0001. **(b-c)** Gene

ontology (GO) analysis of downregulated proteins ($p<0.05$, average log2<-0.6) after 48 hours TM treatment in LM2 SOX2/OCT4+ GFP+ and LM2 SOX2/OCT4- GFP- cells (n=5 samples/group). log_2FC is log2 fold change of TM 48 hour/Control. Count refers to how many genes/proteins showed up in that GO term. GO analysis was performed using the clusterProfiler R package, p values were adjusted using Benjamin-Hochberg method.

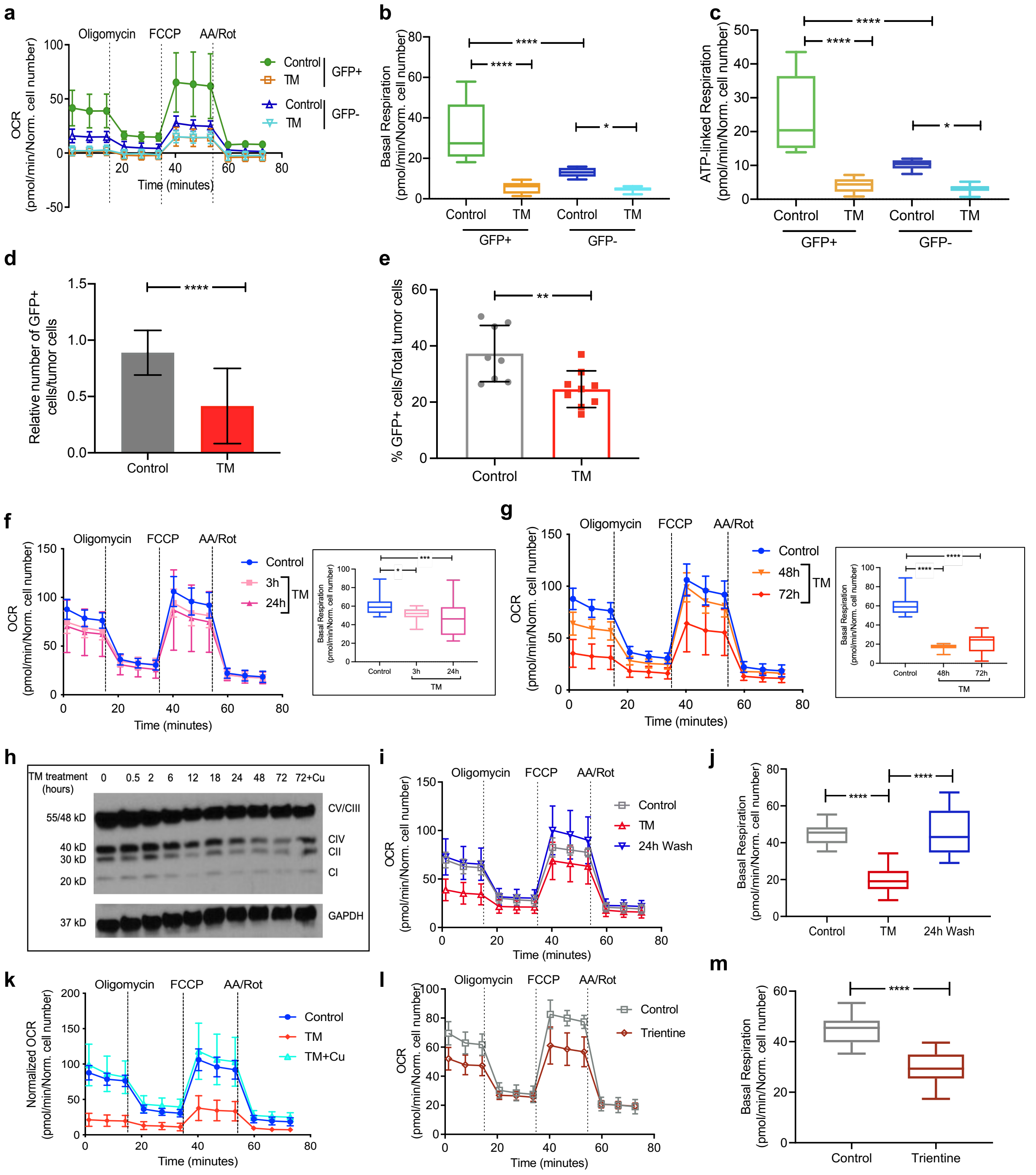

Supplementary Figure 3. (a) Oxygen consumption rate of sorted GFP+ and GFP- populations from LM2 cells (n=4/group). Basal respiration **(b)** and ATP-linked respiration **(c)** of GFP+ vs. GFP- LM2 cells with and without TM. Significance was calculated using One-way ANOVA with Tukey post-test for comparing multiple groups. Center lines of box plots denote median values,

top whiskers denote maxima, and bottom whiskers minima. **(d)** Relative number of GFP+ cells per total tumor cells. (n=5 samples/group, IF images from fig 4e, quantified from 9 fields of view per sample). Analysis was performed using Mann-Whitney two-sided test. **(e)** Impact of TM (0.7 mg/day) (n=9) on percentage of GFP+ cells in ML1-derived primary tumors *in vivo* compared to controls (n=8), all tumor cells expressed mCherry by flow cytometry. Analysis was performed by unpaired two-sided t-test. p-value = 0.0068. **(f-g)** Cellular bioenergetics were determined in control vs. TM (0.5 μM) treated LM2 cells at 3, 24, 48 and 72 hours (n=8 for controls, 3 h, 24 h, and 48 h TM treatments, n=32 for 72 h TM treatment). Quantified basal respiration is represented in the right panel. Significance was calculated using Kruskal-Wallis test. Center lines of box plots denote median values, top whiskers denote maxima, and bottom whiskers minima. **(h)** Effect of TM treatment on ETC complexes over a 72-hour time-period, and with copper rescue. Complexes I-V are referred to as CI (mol.wt.~19 kD), CII (mol.wt.~30 kD), CIII (mol.wt.~48 kD), CIV (mol.wt.~40kD), & CV (mol.wt.~55 kD). **(i)** Effect of TM removal (24 hours of TM removal after 48 hours of treatment with 0.5 μM TM) from treated LM2 cells on oxygen consumption (n=4 for controls, n=8 for TM and 24-hour wash). **(j)** Quantification of basal respiration rate after TM wash (24 hours). Significance was calculated using One-way ANOVA with Tukey post-test for comparing multiple groups. Center lines of box plots denote median values, top whiskers denote maxima, and bottom whiskers minima. **(k)** $CuCl_2$ (0.5 μM) was added back to TM treated LM2 samples for the 24 hours before mitostress assay to rescue oxygen consumption with TM treatment (n=8/group). **(l)** Effect of another copper chelator, trientine (50 μM), on oxygen consumption for LM2 cells (n=4 for controls, n=8 for trientine). **(m)** Quantification of basal respiration with another copper chelator, trientine (50 μM), after 72h of treatment. Analysis was performed by unpaired two-sided t-test. Center lines of box plots denote median values, top whiskers denote maxima, and

bottom whiskers minima. Representative data of two independent experiments are depicted. Results are expressed as mean ± SD. *p<0.05, **p<0.01, ****p<0.0001

a

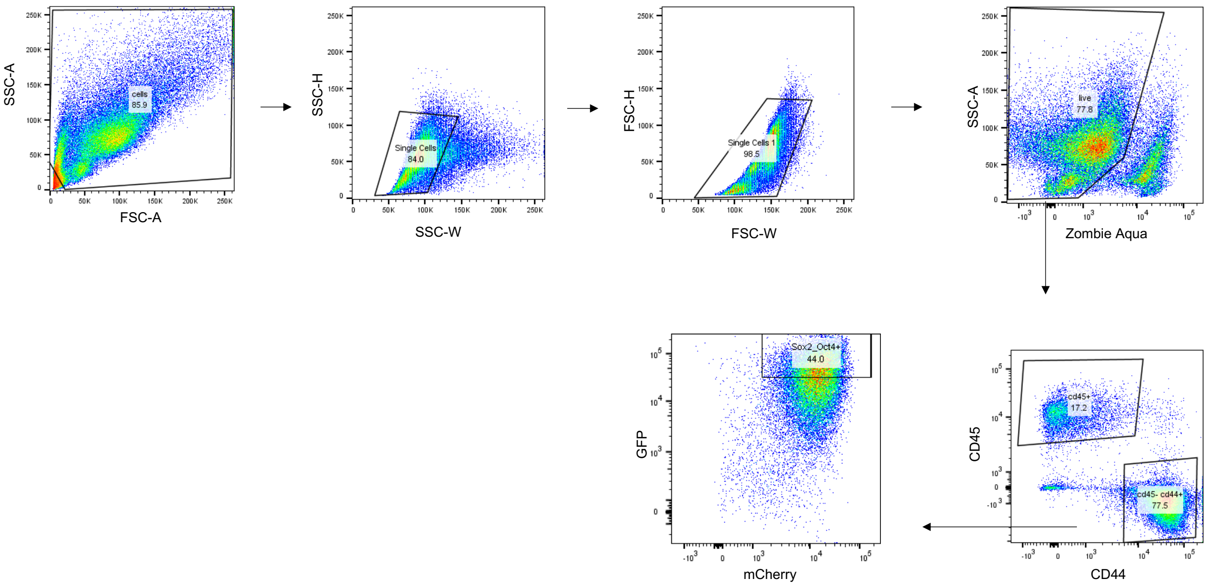

b

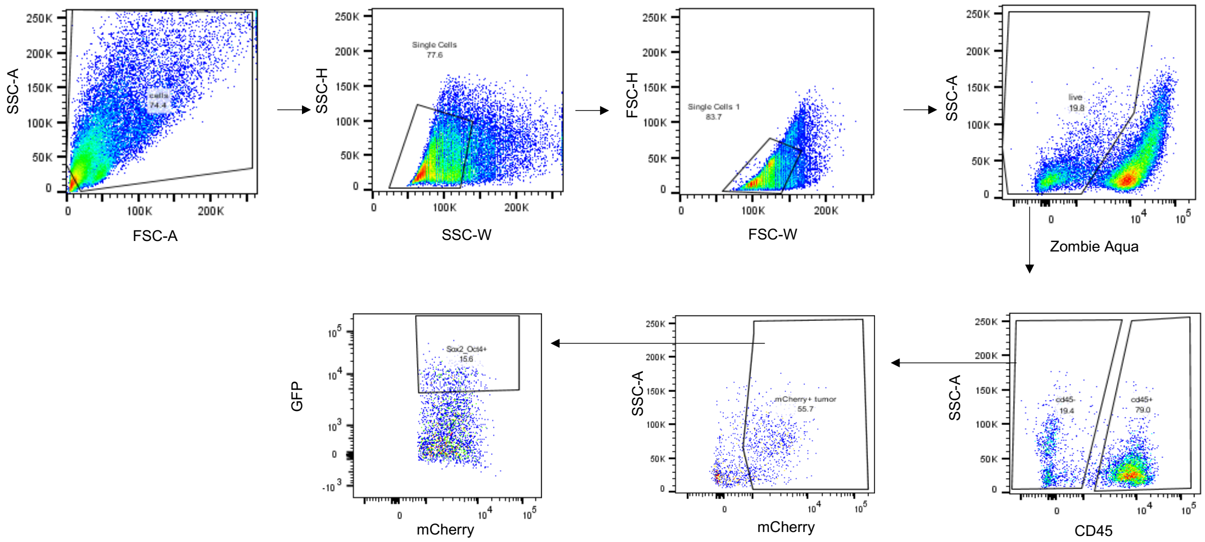

Supplementary Figure 4. (a) Gating strategy for LM2 *in vivo* model. **(b)** Gating strategy for ML1 *in vivo* model.

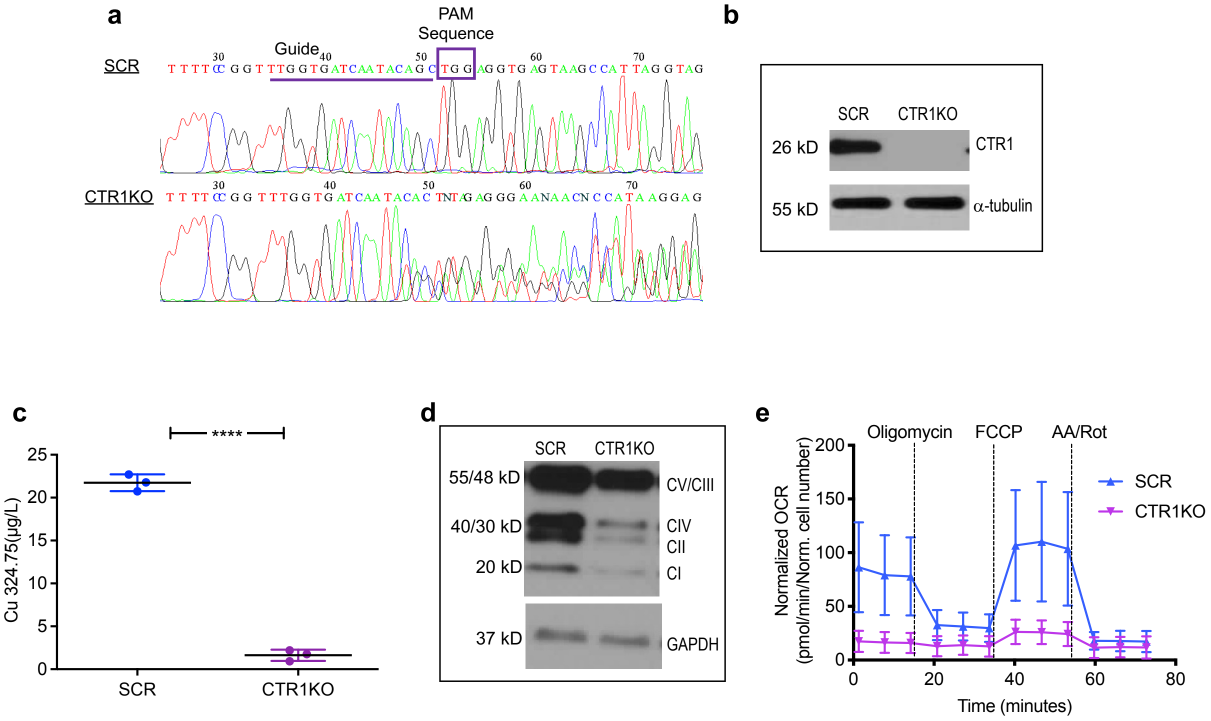

Supplementary Figure 5. (a) Human CTR1 guide and PAM sequence in SCR vs CTR1KO. **(b)** Western blot showing CRISPR-mediated knockout of CTR1 in LM2 cells, SCR is scrambled control. **(c)** Intracellular copper levels as determined by GF-AAS (y-axis refers to cooper content measured at 324.75 nm wavelength). Analysis was performed by unpaired two-sided t-test. **(d)** Western blot showing ETC complexes in SCR vs. CTR1KO. Complexes I-V are referred to as CI (mol.wt.~19 kD), CII (mol.wt.~30 kD), CIII (mol.wt.~48 kD), CIV (mol.wt.~40kD), & CV (mol.wt.~55 kD). **(e)** Oxygen consumption rate in CTR1KO as compared to scrambled control (n=8/group). Representative data of two independent experiments are depicted. Results are expressed as mean ± SD. **p<0.01

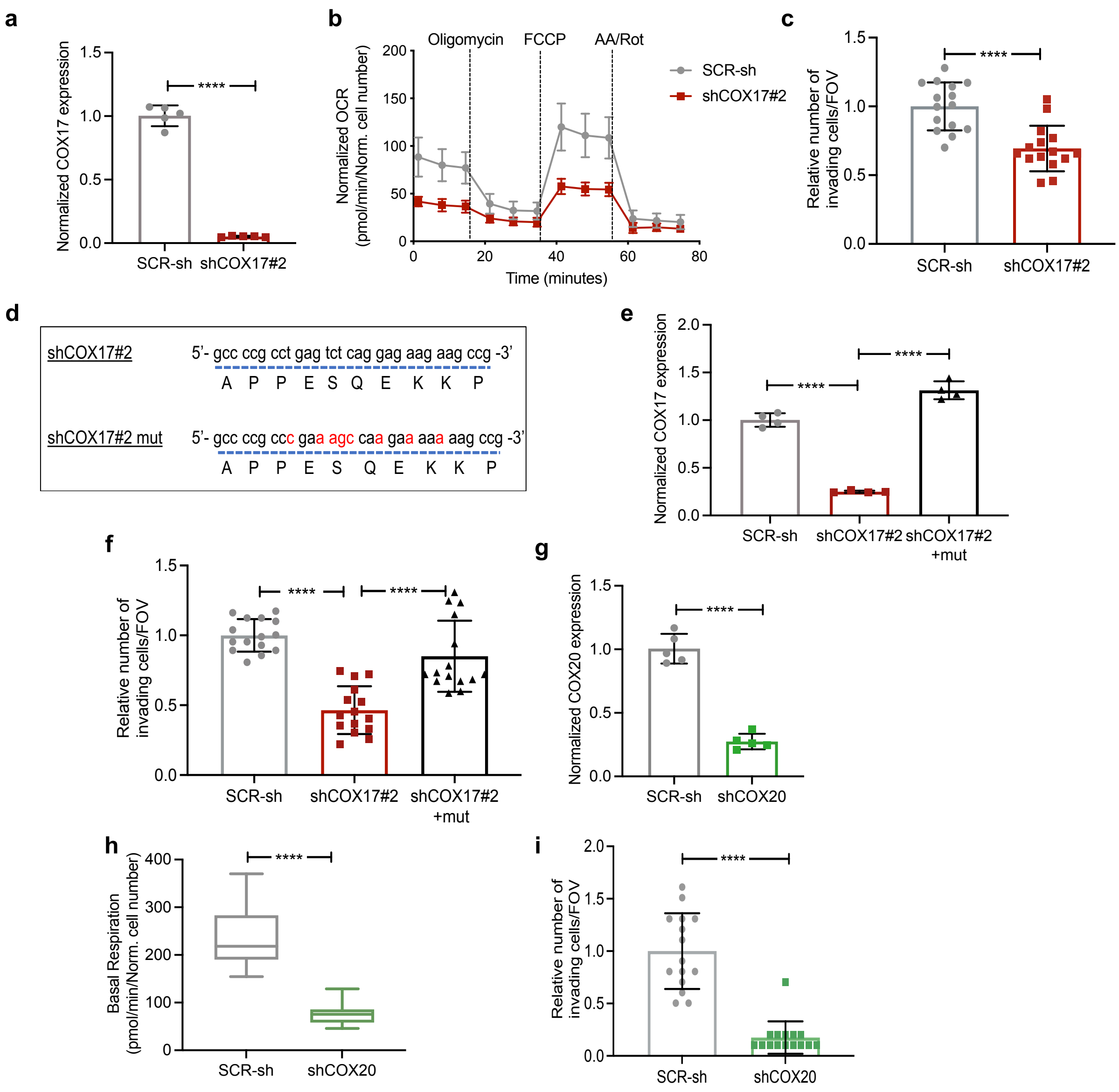

Supplementary Figure 6. (a) qPCR showing COX17 knockdown with a second shRNA (n=5/group) in LM2 cells. Analysis was performed by unpaired two-sided t-test (n=5/group). **(b)** Oxygen consumption in second COX17 hairpin (n=8 for SCR-sh, n=4 for shCOX17#2). **(c)** Invasion assay through matrix coated transwell using second COX17 hairpin. Analysis was performed by unpaired two-sided t-test (n=3/group, 5 fields of view/sample). **(d)** Mutant sequence for COX17 hairpin#2. **(e)** COX17 expression in scrambled, COX17 knockdown and COX17 knockdown cells with transient transfection of COX17 mutant. Significance was calculated using

One-way ANOVA with Tukey post-test for comparing multiple groups (n=4/group). **(f)** Rescue of invasion after transfecting mutant COX17 in cells expressing shCOX17#2. Analysis was performed by one-way ANOVA (n=3/group, 5 fields of view/sample). **(g)** qPCR showing shRNA-mediated COX20 knockdown in ML1 cells (n=5/group). Analysis was performed by unpaired two-sided t-test. **(h)** Basal respiration in ML1 SCR-sh vs. shCOX20 cells (n=8/group shCOX20). Analysis was performed by unpaired two-sided t-test. Center lines of box plots denote median values, top whiskers denote maxima, and bottom whiskers minima. **(i)** Invasion assay through matrix coated transwell. Analysis was performed by Mann-Whitney two-sided test (n=3/group; 5 fields of view/sample). Representative data of two independent experiments are depicted. Results are expressed as mean ± SD. *$p<0.05$, **$p<0.01$, ****$p<0.0001$

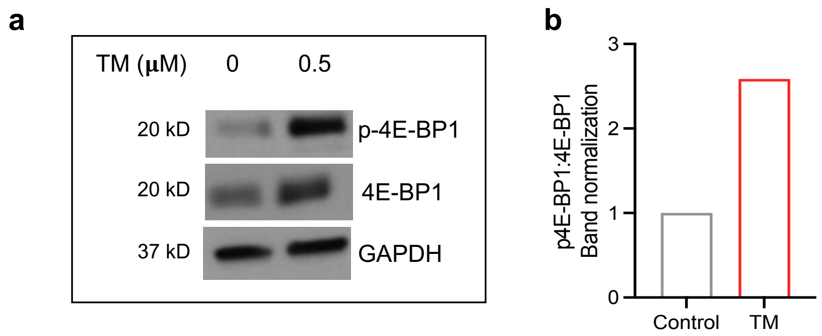

Supplementary Figure 7. (a) Expression of 4E-BP1 after 72-hour TM (0.5 µM) in LM2 cells. **(b)** Quantification of western blot band area using ImageJ software. Representative data of two independent experiments are depicted.
