# Supplementary material for: Copper depletion modulates mitochondrial oxidative phosphorylation to impair triple negative breast cancer metastasis
Source: Nat Commun. 2021 Dec 15;12:7311. doi: 10.1038/s41467-021-27559-z (PMC8674260; doi:10.1038/s41467-021-27559-z)
Supplement: Supplementary file 2 — Reporting summary [file 41467_2021_27559_MOESM2_ESM.pdf]

nature research

Corresponding author(s): Linda Vahdat and Vivek Mittal

Last updated by author(s): Oct 15, 2021

Reporting Summary

Nature Research wishes to improve the reproducibility of the work that we publish. This form provides structure for consistency and transparency in reporting. For further information on Nature Research policies, see our Editorial Policies and the Editorial Policy Checklist.

Statistics

For all statistical analyses, confirm that the following items are present in the figure legend, table legend, main text, or Methods section.

n/a	Confirmed	
☐	☒	The exact sample size (*n*) for each experimental group/condition, given as a discrete number and unit of measurement
☐	☒	A statement on whether measurements were taken from distinct samples or whether the same sample was measured repeatedly
☐	☒	The statistical test(s) used AND whether they are one- or two-sided<br>*Only common tests should be described solely by name; describe more complex techniques in the Methods section.*
☒	☐	A description of all covariates tested
☐	☒	A description of any assumptions or corrections, such as tests of normality and adjustment for multiple comparisons
☐	☒	A full description of the statistical parameters including central tendency (e.g. means) or other basic estimates (e.g. regression coefficient) AND variation (e.g. standard deviation) or associated estimates of uncertainty (e.g. confidence intervals)
☐	☒	For null hypothesis testing, the test statistic (e.g. *F*, *t*, *r*) with confidence intervals, effect sizes, degrees of freedom and *P* value noted<br>*Give P values as exact values whenever suitable.*
☒	☐	For Bayesian analysis, information on the choice of priors and Markov chain Monte Carlo settings
☐	☒	For hierarchical and complex designs, identification of the appropriate level for tests and full reporting of outcomes
☒	☐	Estimates of effect sizes (e.g. Cohen's *d*, Pearson's *r*), indicating how they were calculated

Our web collection on statistics for biologists contains articles on many of the points above.

Software and code

Policy information about availability of computer code

Data collection	Mus musculus/ Homo sapiens UniProt protein database, downloaded on 2019/12/13<br>MassHunter Metabolomics dMRM Database and Method
Data analysis	RStudio v1.3<br>Proteome Discoverer (PD) version 2.4.1.15<br>MassHunter Workstation Software for Quantitative analysis B.09<br>Bio-rad CFX Maestro version 4.1.2434.0124<br>Seahorse Wave Desktop Software v2.6.1<br>Graphpad Prism v8 and v9<br>Aperio ImageScope v12.4.3.5008<br>ImageJ1.52k<br>FlowJo10.6.2

For manuscripts utilizing custom algorithms or software that are central to the research but not yet described in published literature, software must be made available to editors and reviewers. We strongly encourage code deposition in a community repository (e.g. GitHub). See the Nature Research guidelines for submitting code & software for further information.

Data

Policy information about availability of data

All manuscripts must include a data availability statement. This statement should provide the following information, where applicable:

- Accession codes, unique identifiers, or web links for publicly available datasets
- A list of figures that have associated raw data
- A description of any restrictions on data availability

The authors declare that the data supporting the findings of this study are available within the paper [and its Supplementary Information files]. Source data are provided with this paper. Raw proteomics data have been uploaded to ProteomeXchange.org (Project accession: PXD027089). Other datasets used in the study include: METABRIC (EGAS00001001753) and Phase II clinical trial of TM (NCT00195091) (Chan et al., 2017). Any additional detail can be requested from the corresponding authors.

Field-specific reporting

Please select the one below that is the best fit for your research. If you are not sure, read the appropriate sections before making your selection.

☒ Life sciences ☐ Behavioural & social sciences ☐ Ecological, evolutionary & environmental sciences

For a reference copy of the document with all sections, see nature.com/documents/nr-reporting-summary-flat.pdf

Life sciences study design

All studies must disclose on these points even when the disclosure is negative.

Sample size	For in vitro experiments, n numbers of each group were indicated as for biologic replicates in the figure legends. For in vivo experiments, each cohort comprises n>=5 mice per treatment group. Based on prior mouse experiments, this number will provide general power to detect differences greater than 50% at a significance of p<0.05 (Ban et al. doi: 10.1038/s43018-021-00245-1). Only for in vivo metastasis assay (Fig. 1h), we had n=4 mice per group. This was due to either mouse death during surgery or Research Animal Resource Center (RARC) recommended euthanasia due to ulcerated/infected primary tumors. The experiment was replicated independently (information provided in source data).
Data exclusions	Any outlier, if at all, was excluded after analyzing the datasets with ROUT method. This was done for Fig. 5g.
Replication	Every experiment was repeated at least twice, either with the same cell line, or another TNBC cell line or both. All attempts of verification of experiment findings were successful.
Randomization	No randomization was used. 8 week old female mice were randomly chosen for control or treated cohorts in the experiments as described in the text and Methods section (Mouse strains).
Blinding	Investigators were not blinded during in vivo or in vitro experiments because the investigator who participated in experimental design, also performed the experiment.

Reporting for specific materials, systems and methods

We require information from authors about some types of materials, experimental systems and methods used in many studies. Here, indicate whether each material, system or method listed is relevant to your study. If you are not sure if a list item applies to your research, read the appropriate section before selecting a response.

Materials & experimental systems

n/a	Involved in the study	
☐	☒	Antibodies
☐	☒	Eukaryotic cell lines
☒	☐	Palaeontology and archaeology
☐	☒	Animals and other organisms
☒	☐	Human research participants
☐	☒	Clinical data
☒	☐	Dual use research of concern

Methods

n/a	Involved in the study	
☒	☐	ChIP-seq
☐	☒	Flow cytometry
☒	☐	MRI-based neuroimaging

Antibodies

Antibodies used	Total OXPHOS Rodent WB Antibody Cocktail Abcam Cat# ab110413, RRID:AB_2629281 (1:250)<br>NDUFA4 Bioworld antibodies Cat# BS3883, RRID:AB_1914279 (1:500)

	AMPK alpha Cell signaling technologies Cat# 5831S, RRID:AB_10622186 (1:1000)<br>Phospho-AMPKα (Thr172) Cell signaling technologies Cat# 50081S, RRID:AB_2799368 (1:1000)<br>Acetyl-CoA Carboxylase Antibody Cell signaling technologies Cat# 3662S, RRID:AB_2219400 (1:1000)<br>Phospho-Acetyl-CoA Carboxylase (Ser79) Antibody Cell signaling technologies Cat# 3661S, RRID:AB_330337 (1:1000)<br>Raptor Cell signaling technologies Cat# 2280S, RRID:AB_561245 (1:1000)<br>Phospho-Raptor (Ser792) Cell signaling technologies Cat# 2083S, RRID:AB_2249475 (1:1000)<br>p70 S6 Kinase Cell signaling technologies Cat# 9202S, RRID:AB_331676 (1:1000)<br>Phospho-p70 S6 Kinase (Thr421/Ser424) Cell signaling technologies Cat# 9204S, RRID:AB_2265913 (1:1000)<br>CTR1 Cell signaling technologies Cat# 13086S, RRID:AB_2798116 (1:1000)<br>α-tubulin Proteintech Cat# 11224-1-AP, RRID:AB_2210206 (1:1000)<br>GAPDH Abcam Cat# ab9485, RRID:AB_307275 (1:2000)<br>Anti-mouse CD45, clone 30-F11 Biolegend Cat# 103134, RRID:AB_2562559 (1:100)<br>Anti-human CD44, clone BJ18 Biolegend Cat# 338816, RRID:AB_2716001 (1:100)<br>Rat IgG HRP-conjugated Antibody R&D Cat#HAF005, RRID:AB_1512258 (1:5000)<br>anti-mouse IgG -HRP conjugate R&D Cat# HAF007, RRID:AB_357234 (1:5000)
Validation	The commercial antibodies are well used and reported in previous publications. All primary antibodies used have been verified for species specificity as described on the manufacturer's website.

Eukaryotic cell lines

Policy information about cell lines

Cell line source(s)	MDA-MB-231.LM2 (Minn et al., 2005), MTA with Memorial Sloan Kettering Cancer Center, New York (Dr. Joan Massagué)<br>MDA-MB-231.LM2 CTR1KO This Paper<br>MDA-MB-231.LM2 SCR This Paper<br>MDA-MB-231.LM2 scr-sh This Paper<br>MDA-MB-231.LM2 shCOX17#1 This Paper<br>MDA-MB-231.LM2 shCOX17#2 This Paper<br>EO771 (Johnstone et al., 2015), obtained from Dr. Robin Anderson (La Trobe University, Heidelberg Vic Australia)<br>EO771.ML1 This Paper<br>EO771.ML1 shCOX20 This Paper<br>EO771.ML1 scr-sh This Paper<br>MDA-MB-468 ATCC HTB-132<br>HEK-293T ATCC CRL-11268
Authentication	All primary cell lines were authenticated in previous publications. Cell lines generated in this study have been authenticated using species-specific primers.
Mycoplasma contamination	All cell lines were routinely tested for mycoplasma contamination. All cell lines used were negative for mycoplasma but not described in text.
Commonly misidentified lines<br>(See ICLAC register)	No Commonly misidentified lines were used.

Animals and other organisms

Policy information about studies involving animals; ARRIVE guidelines recommended for reporting animal research

Laboratory animals	CB-17 SCID Charles River (236)<br>C57BL/6J Jackson Laboratory (000664)<br>All mice used in this study were 8-weeks old, female.
Wild animals	Study did not involve wild animals.
Field-collected samples	Study did not involve field-collected samples
Ethics oversight	All animal studies were approved by the Institutional Animal Care and Use Committee at Weill Cornell Medical College.

Note that full information on the approval of the study protocol must also be provided in the manuscript.

Clinical data

Policy information about clinical studies

All manuscripts should comply with the ICMJE guidelines for publication of clinical research and a completed CONSORT checklist must be included with all submissions.

Clinical trial registration	NCT00195091
Study protocol	Phase II clinical trial of TM (NCT00195091) (Chan et al., 2017). Full protocol is available from the corresponding author upon request.
Data collection	Patients were accrued between June 2007 and August 2014 and follow-up continued through Jan 2020.
Outcomes	The primary endpoint was to assess the change in VEGFR2.<br>EPCs in patients who were treated with oral tetrathiomolybdate<br>for 2 years. Secondary endpoints were safety, event-free survival<br>(EFS), effect of tetrathiomolybdate on VEGFR1. HPCs, and levels<br>of plasma angiogenic factors and cytokines.<br>Imaging every 6 mos or as needed to assess for relapse, using Response<br>Evaluation Criteria in Solid Tumors (RECIST)<br>Adverse event/toxicity assessments were assessed continually using CTCAE v3.0

Flow Cytometry

Plots

Confirm that:

- [x] The axis labels state the marker and fluorochrome used (e.g. CD4-FITC).
- [x] The axis scales are clearly visible. Include numbers along axes only for bottom left plot of group (a 'group' is an analysis of identical markers).
- [x] All plots are contour plots with outliers or pseudocolor plots.
- [x] A numerical value for number of cells or percentage (with statistics) is provided.

Methodology

Sample preparation	For flow cytometry analysis, tissues (primary tumor or lungs) were diced, ground through a 140 µm mesh, filtered through a 70 µm filter, red blood cells were lysed, followed by live/dead staining using Zombie Aqua, and Fc blocked and surface stained with human CD44 (clone BJ18) and mouse CD45 (clone 30-F11) antibodies. Disseminated tumor cells in the lungs were identified as CD45-CD44+mCherry+ per 106 live cells. Human LM2 and mouse ML1 cells stably expressing the SOX2/ OCT4-GFP promoter reporter were identified by FACS as CD45- CD44+ mCherry+ GFP+ and CD45- mCherry+ GFP+, respectively. Analysis of flow cytometry data was performed using FlowJo v 10.6.2.
Instrument	Becton-Dickinson Fortessa Analyzer
Software	BD FACSDiva, FlowJo10.6.2
Cell population abundance	Tumor and myeloid cell population abundance in dissociated tissue samples was confirmed with cell surface antibodies (CD45 for myeloid cell, CD44 for LM2 cells) or fluorescent markers (mCherry or GFP).
Gating strategy	Cells were first gated based on FSC-A vs. SSC-A. Single cells were then selected (FSC-W vs. FSC-H and SSC-W vs. SSC-H), followed by selection of live cells using Zombie Aqua. The cells were then gated as described in the methods.

- [x] Tick this box to confirm that a figure exemplifying the gating strategy is provided in the Supplementary Information.
